# Discovering de novo peptide substrates for enzymes using machine learning

**DOI:** 10.1038/s41467-018-07717-6

**Published:** 2018-12-07

**Authors:** Lorillee Tallorin, JiaLei Wang, Woojoo E. Kim, Swagat Sahu, Nicolas M. Kosa, Pu Yang, Matthew Thompson, Michael K. Gilson, Peter I. Frazier, Michael D. Burkart, Nathan C. Gianneschi

**Affiliations:** 1 Department of Chemistry and Biochemistry, University of California San Diego, 9500 Gilman Drive, La Jolla, CA 92093-0358 USA; 2 School of Operations Research and Information Engineering, Cornell University, 232 Rhodes Hall, Ithaca, NY 14853-3801 USA; 3 Department of Chemistry, Department of Materials Science and Engineering, Department of Biomedical Engineering, Northwestern University, 2145 Sheridan Road, Evanston, IL 60208 USA; 4 Skaggs School of Pharmacy and Pharmaceutical Sciences, University of California San Diego, 9500 Gilman Drive, La Jolla, CA 92093 USA

## Abstract

The discovery of peptide substrates for enzymes with exclusive, selective activities is a central goal in chemical biology. In this paper, we develop a hybrid computational and biochemical method to rapidly optimize peptides for specific, orthogonal biochemical functions. The method is an iterative machine learning process by which experimental data is deposited into a mathematical algorithm that selects potential peptide substrates to be tested experimentally. Once tested, the algorithm uses the experimental data to refine future selections. This process is repeated until a suitable set of de novo peptide substrates are discovered. We employed this technology to discover orthogonal peptide substrates for 4’-phosphopantetheinyl transferase, an enzyme class that covalently modifies proteins. In this manner, we have demonstrated that machine learning can be leveraged to guide peptide optimization for specific biochemical functions not immediately accessible by biological screening techniques, such as phage display and random mutagenesis.

## Abstract (web summary)

The discovery of peptide substrates for enzymes with selective activities is a central goal in chemical biology. Here, the authors develop a hybrid method combining machine learning and experimental testing for fast optimization of peptides for specific, orthogononal functions.

## Introduction

Machine learning has garnered increased attention in recent years for success in applications ranging from internet commerce to autonomous vehicles^1–5^ due in large part to advances in computing power and availability of data^6^. Machine learning methods enable the best selection among a set of diverse options^7,8^ by predicting their quality. They can also identify which additional experimental data would best improve prediction^9^. This process affords a more efficient and economical approach than unguided experimental evaluation. While application of machine learning towards the optimization and discovery of biochemical systems promises success similar to that observed in business and engineering design problems^9–11^, there have been relatively few biochemical applications reported for optimizing multiple parameters in parallel^12–15^. Here, we highlight a machine learning method that enables the discovery of selective substrates for a set of functionally related enzymes, where little orthogonality exists in nature.

Previous biochemical applications of machine learning used the pure-exploitation approach^16^, in which confirmatory assays experimentally evaluate only those options with the best predicted performance^12,17,18^. More sophisticated Bayesian optimization^9,10^ and optimal learning^16^ approaches to designing assays given a machine learning-based predictive model have been shown to better assist in optimizing systems using a limited number of experimental evaluations^9,19,20^. These approaches experimentally evaluate options with the highest potential to improve over the best previously evaluated option^21^. While previous work proposes Bayesian optimization for biological molecular design applications^13,15,22^, there is a current interest for implementing machine learning toward macromolecular optimization and small-molecule discovery^12–15^.

Using this optimal learning approach, we developed a method entitled Peptide Optimization with Optimal Learning (POOL) to identify short (8–20 residues) peptides as selective substrates for enzymes. To validate POOL, we used a post-translational modifying enzyme, the 4′-phosphopantetheinyl transferase (PPTase)^23^. PPTases covalently modify carrier proteins (CPs) involved in various biosynthetic pathways at a conserved serine residue by the addition of phosphopantetheine derived from coenzyme A (CoA) (Supplementary Figure 1A–C)^23^. Previous work on PPTases using phage display led to the discovery of the 11-residue peptide, YbbR, that can act as a surrogate for the full-length CP and be used as a short peptide tag for such applications as biochemical protein labeling and affinity purification^24^.

Here, we used POOL to guide our experimental high-throughput cellulose SPOT synthesis screening array^25^ to identify short peptides that meet the following criteria: (1) enzymatic activity by PPTases by covalent attachment (labeling) of CoA analog to peptide substrate; (2) orthogonal, in which a given peptide is a substrate for one class of PPTase, but not the other; these PPTase classes include the Sfp-type (surfactin phosphopantetheinyl transferase from *Bacillus subtilis*) and AcpS-type (*holo*-acyl carrier protein synthase from *Streptomyces coelicolor*) (Supplementary Figure 1B). Due to their structural difference and nature of interaction between the various CPs, the pseudodimer, Sfp, is known to be promiscuous toward a variety of CPs, while the homotrimer, AcpS, is more selective^23^. In this study, we demonstrate how POOL addresses complex biochemical challenges that are difficult or impossible to solve by conventional methods to identify active orthogonal peptide substrates for multiple post-translational modification enzymes.

We demonstrate a computationally driven machine learning system, POOL, that guides the evolution of optimized orthogonal peptide substrates by alternating between prediction and targeted experimentation. This targeted approach departs from methods that randomly generate and experimentally screen many peptides, and can optimize for multiple complex biochemical activities, such as the ability to be selective for specific enzymes and undergo chemical transformations that would normally require several tandem screens to achieve. We show that POOL efficiently identifies both active and orthogonal peptide substrates for post-translational modification enzymes that are uniquely diverse from its original training set. POOL increases its chance of identifying active substrates by: (1) combining information across enzymes with a predictive model; (2) diversifying selections against prediction uncertainty, using ideas from Bayesian optimization; (3) incorporating feedback iteratively. POOL can be applied generally to optimize peptides for specific and/or simultaneous biochemical activity.

## Results

### Hits identified by POOL

**Fig. 1:**
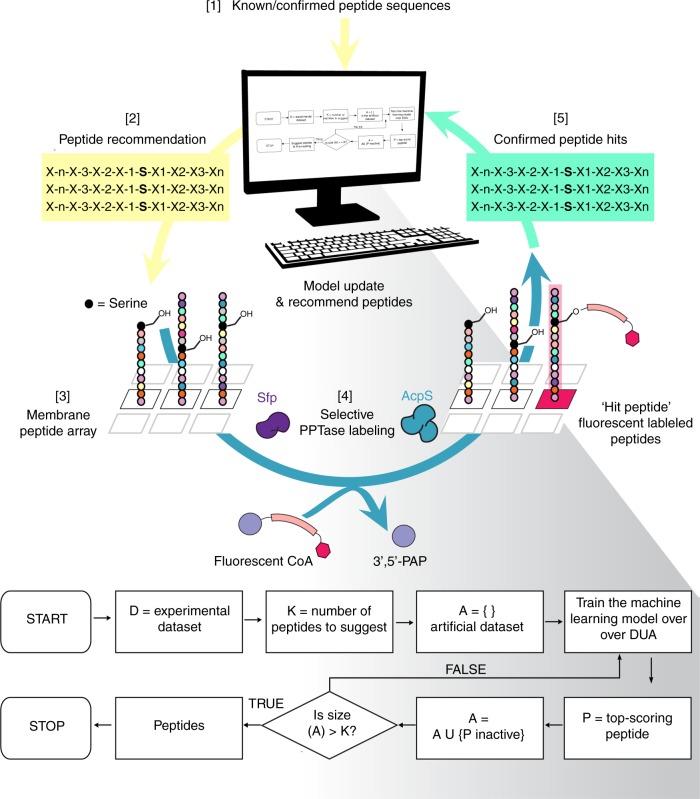
Overview of the iterative Peptide Optimization with Optimal Learning (POOL) method workflow. Top cycle illustrates the autonomous computer algorithm coupled with experimental testing of peptide recommendations. [1] An initial set of peptides from known substrates of PPTase (Supplementary Methods 1) are entered in the algorithm defined in the box below. [2] A set of peptides are recommended. [3] Next, peptides are synthesized on a cellulose membrane where peptides are [4] chemoenzymatically labeled by PPTase (Sfp or AcpS) (hit peptide labeled in pink) (PAP is 3′,5′-phosphoadenosine phosphate). [5] Experimentally confirmed peptide hits are fed back into the algorithm and the process repeats. The details of the algorithm used to recommend peptides are described in Supplementary Methods 2

Truncated portions of the acyl carrier protein (ACP) and peptides known to be active for either Sfp or AcpS^24,26^ along with additional truncated proteins and peptides known to be inactive were initially used to train the POOL model to generate predicted short (8–20 amino acid) peptide substrates. The POOL model produces a single top-scoring short peptide from this initial data. To decrease statistical overfitting, the natural 20 amino acid residues were organized into eight reduced residue classes by their chemical properties (Supplementary Figure 2). Prior to any experimental confirmation, the algorithm calculates a new prediction model based on the same set of initial experimental data used to generate this top-scoring peptide, but with the assumption that the top-scoring peptide is not a hit; that is, it lacks the desired pattern of orthogonal activity. POOL subsequently selects a second top-scoring peptide based on this new model. Then, a third model is produced based on the second experimental data, with an assumption that neither of the first two peptides are hits and another top-scoring peptide from this third model is chosen. This process iterates so that each successive peptide is chosen based on a model that uses the existing experimental data and the assumption that all previously chosen peptides in the present set are not hits, until the desired number of peptides to be tested is reached (Fig. 1). The assumption that previously recommended peptides are not hits in these successive models captures the real-world possibility that the predictions of any given model are not valid and causes subsequent newly recommended peptides to be different from previously recommended ones, diversifying the set of peptides tested while still including those likely to be a hit.

POOL was designed to find short hits in a small number of experimental testing rounds to satisfy our criteria. Experimental confirmation of predicted peptides was conducted by the synthesis of membrane arrays containing 600 peptides. The peptides were synthesized and displayed on a cellulose membrane and then screened for enzymatic activity. These SPOT membranes were evaluated for orthogonal labeling activity by using fluorescent CoA^27^ to identify peptides labeled by two different PPTases (Sfp and AcpS) (Fig. 1). After one round, we identified peptides that were both hits and misses and inputted these to the POOL algorithm for analysis. The POOL algorithm then recommended a new set of peptides to test in the subsequent round. This process was repeated for four rounds to identify de novo peptide substrates.

**Fig. 2:**
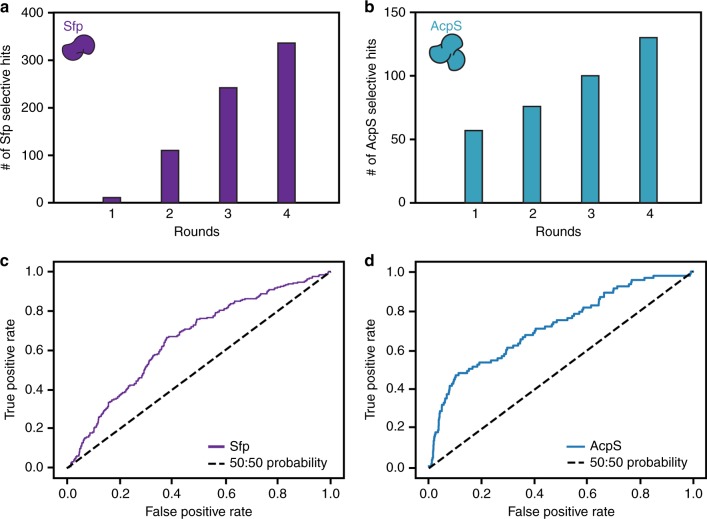
Specific peptide hits identified by POOL and model validation. The number of peptides with specific labeling for Sfp (purple bar graph) (**a**) and AcpS (cyan bar graph) (**b**) over four rounds. The receiver operating characteristic (ROC) curve plot for both Sfp (purple curve) (**c**) and AcpS (cyan curve) (**d**) selective labeling, which diagnoses POOL’s ability to predict hit peptide substrates. The dashed line is the baseline that indicates the performance of a hypothetical random model without prediction power

Initial rounds of Sfp labeling yielded significantly more peptide substrates (hits) than AcpS, presumably because our preliminary experimental data came primarily from known protein targets of Sfp. Iterative cycles of experimental input from SPOT membrane treatments with orthogonal PPTases with POOL showed an increase in the number of peptide hits (i.e., orthogonal substrates for Sfp and AcpS, Fig. 2a, b) after several rounds.

Importantly, screening of the initial predicted Sfp-specific peptides garnered less than five hits, while the last round of Sfp peptides yielded a 10-fold increase in peptide hits (Fig. 2a).

### Model validation

To evaluate the accuracy of the prediction model within POOL, receiver operating characteristic (ROC) curves (Fig. 2c, d), which are commonly used to evaluate predictive models for classification, were generated from peptide hits data. The dashed straight line in each plot is the baseline, indicating the performance of a hypothetical random model without prediction power. The further the solid curved line trends away from the baseline toward the upper left corner, the higher the prediction accuracy exhibited by the model.

As expected, the trained prediction model within POOL provided predictions that were substantially better than random chance, but still imperfect. These imperfections are due to four factors. First, POOL's prediction model uses a reduced amino acid alphabet that does not distinguish between amino acids in the same class. Second, POOL’s predictive model makes a conditional independence assumption. That is, that an amino acid’s contribution to activity at a position does not depend on the amino acids at other positions. This reduces the amount of data required to make predictions but introduces statistical bias. Third, predictive performance is evaluated on peptides recommended by POOL, which were selected in part because they were likely to be active. Ordering these by activity level is substantially more challenging than it would be for peptides with a wider range of predicted activity. Fourth and finally, the quantity of initial experimental data is small relative to the complexity of the space of peptides over which prediction was performed. These factors are discussed in more detail in the supplementary information (Supplementary Methods 2B).

A traditional pure-exploitation approach for selecting peptides based on a predictive model with such imperfect accuracy would have had limited success because of sensitivity to prediction error during the discovery phase (Supplementary Methods 3B). Unlike this traditional approach, POOL accounts for prediction uncertainty and actively diversifies its selections to increase the chance of finding hits, as discussed below.

### POOL performance relative to other methods evaluated via simulation

The POOL method first analyzes existing experimental data to produce an initial machine learning model to predict peptide substrates that are selective for each PPTase type. The POOL approach then chooses experiments to perform that are likely to reveal hits, even when a lack of training data creates less than perfect predictive accuracy within an iterative scheme. Unlike a pure-exploitation or predict-then-optimize method (Supplementary Methods 3A) which does not explicitly account for prediction error, the POOL approach contains a built-in contingency plan to combat prediction errors in previously added peptides, while also broadening the diversity of potential peptide substrates (Fig. 1). This mirrors the importance of balancing exploitation (selecting options predicted to work well) and exploration (selecting items with uncertain predictions) found in other applications of Bayesian optimization^16^.

**Fig. 3:**
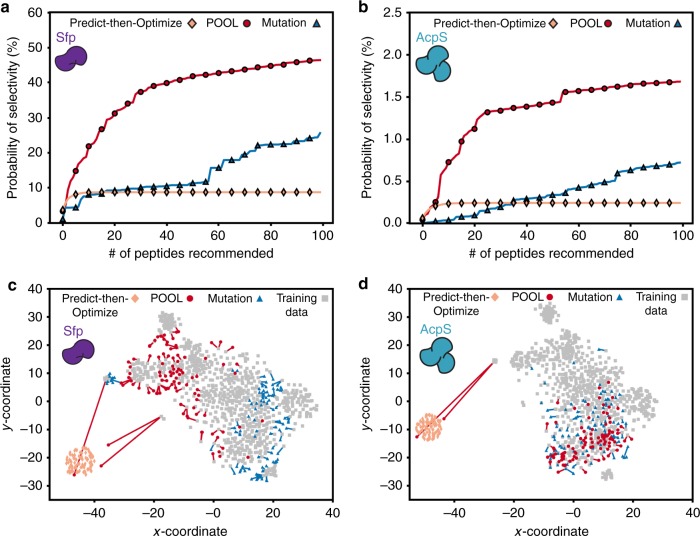
Simulated method performance comparison and peptide diversity evaluation. A comparison between the POOL (red circle), mutation (blue triangle), and predict-then optimize (pale orange diamond) methods for Sfp (**a**) and AcpS (**b**) based on a simulation of 100 peptides recommended after observing results from two rounds, and evaluated using four rounds of data. Peptide homology in peptide space for Sfp (**c**) and AcpS (**d**) is shown for each of the methods POOL (red circle), mutation (blue triangle), predict-then-optimize (pale orange diamond) and overlaid with the peptide training data (gray square). Distances between points represents how similar or dissimilar the corresponding peptides are from each other. A line from each peptide recommended by POOL and mutation indicates the closest peptide in the training data

To further confirm this aspect of POOL’s behavior and performance relative to other methods, it was compared via a simulation study with the mutation method and the predict-then-optimize method (Fig. 3, Supplementary Methods 3A).

The mutation method takes an evolutionary approach and mutates known hits, while the predict-then-optimize method trains POOL’s machine learning model and recommends peptides predicted to be hits, but does not use POOL’s iterative approach of re-training the model with an artificial dataset. In this simulation study, a peptide’s probability of being a hit was determined via the prediction model trained using data gathered from multiple rounds (2602 peptides). The three methods were then provided with only the initial training set and data from the first two rounds. Then, we asked POOL to recommend 100 new potential Sfp-specific hits and 100 potential AcpS-specific hits. The probability that the recommended set contained at least one peptide hit was then calculated via the trained model for each recommended set. POOL had a higher probability of finding at least one peptide hit in its recommended batch compared to the alternative prediction methods (Fig. 3). This analysis suggests that iterative machine learning of peptide hits over several rounds balancing exploitation and exploration is advantageous as POOL identified multiple, unique orthogonally labeled peptides. The significantly higher probability of finding Sfp-specific hits compared to the other methods in the simulation study suggests that POOL outperformed the other methods for Sfp selectivity (Fig. 3a). Due to the lack of AcpS-specific hits in the training data, all three methods had a low probability of finding AcpS-specific hits, though the POOL method displayed a significantly higher chance of predicting AcpS-selective peptides than mutation and predict-then-optimize (Fig. 3b).

### Diversity of peptides recommended in the simulation study

The simulation study (Fig. 3) suggests that POOL is able to find peptide hits more effectively than the predict-then-optimize and mutation methods because it is able to better balance exploration with exploitation. To further confirm this behavior, we investigated the sequences of the peptides recommended by the different prediction methods in the simulation study, and of the training data. Figure 3c, d shows a two-dimensional (2D) representation of peptides recommended by those methods, where each point in the plot represents a distinct peptide (Supplementary Methods 3C). A large distance between any two points indicates dissimilarity in sequence between the corresponding peptides. Recommended peptides by the predict-then-optimize method clustered together tightly, suggesting that the top-ranking peptides are expected to share similar sequences. Both POOL and the mutation method recommended more diversified peptides than predict-then-optimize. To support comparison between POOL and mutation, Fig. 3c, d includes a line between each of their recommended peptides and the closest peptide in the training data. Lines originating from the mutation method’s peptides tend to be shorter than from POOL’s: mean lengths in Fig. 3c are 1.63 for mutation and 2.55 for POOL, and in Fig. 3d are 0.97 and 1.67, respectively. This is also confirmed by a histogram and additional summary statistics available in the supplementary information (Supplementary Figure 3). This suggests that the process of mutating known hits generates peptides that are more similar to the training data than those generated by POOL. In contrast with mutation, peptides recommended by POOL spread more broadly in 2D space, filling more gaps between peptides in the training data. In addition to this diverse exploratory behavior, POOL chooses at least one peptide in the same region where the predict-then-optimize method recommended a peptide. This pattern indicates that POOL provides a balance between the exploitation of model predictions and diversification accounting for prediction errors.

### Selective and diverse peptide hits identified by POOL

**Fig. 4:**
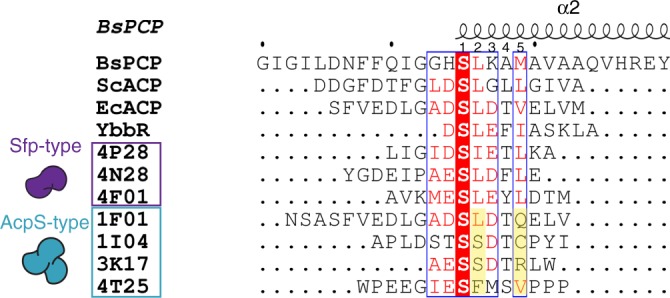
Sequence alignment of hit peptides relative to native PPTase substrates. The sequence alignment for *B. subtilis* PCP, *S. coelicolor* ACP, *E. coli* ACP, YbbR peptide, compared to Sfp-type peptide hits (4P28, 4N28, 4F01) and AcpS-type peptide hits (1F01, 1I04, 3K17, 4T25) to the secondary (α2) structure of *B. subtilis* PCP (PDB: 4MRT). The blue box and red residues show general conserved sequences across all the peptides. The majority of AcpS-type peptides have conserved polar residues in position 2 and 5 (highlighted in yellow). The peptide identification corresponds to the round number and location it was identified from (round number_letter row_spot number on membrane) during the iterative rounds of POOL

To identify sequence elements that appear to confer PPTase selectivity, three Sfp-type and four AcpS-type peptides identified by POOL were aligned to known PPTase substrates. We included in this alignment the *B. subtilis* peptidyl CP (PCP) from the tyrocidine pathway, a known Sfp substrate; *S. coelicolor* ACP and *E. coli* ACP, which are native AcpS-type CPs; and YbbR, a peptide substrate that is recognized by both PPTases (Fig. 4)^23,24,28^.

All substrates recognized by Sfp contain a large hydrophobic residue, here either Ile or Leu, at positions 2 and 5, relative to the catalytic serine (position 1) (Fig. 4). This motif is consistent with a recent crystal structure of a bound complex of PCP with Sfp^28^, where residues 46 and 49 of the PCP, which correspond to positions 2 and 5 here, are Leu and Met, respectively. These residues, located in the helix of the PCP, form favorable contacts with the extended nonpolar patch on Sfp’s catalytic domain^28^. Interestingly, mutation of Met49 in PCP to a polar residue impairs catalytic activity and binding to Sfp^28^. In contrast, the AcpS-type peptides do not show a clear motif involving positions 2 and 5, with Leu, Ser, and Val at position 2, and Gln, Arg, and Val at position 5. These residues presumably disrupt interactions important for binding to Sfp, and thus help account for the AcpS selectivity of these peptides. Perhaps the most consistent feature of these four AcpS substrates is that they have a small hydroxyl residue (Ser or Thr) at position 4 (also present in one of the Sfp substrates).

Sfp is known to be promiscuous toward many CPs from various organisms and biosynthetic pathways, including CP substrates of AcpS^21^. Additionally, CP substrates that are labeled by AcpS, and not Sfp, are rare. POOL successfully identified four AcpS-specific peptides that were not recognized by Sfp, which when compared to the native CP substrates possess highly divergent sequences. This highlights the advantage of POOL over traditional genetic and combinatorial methods. These sequences would not be typically accessible by traditional genetic and combinatorial methods as they are not designed to simultaneously access for orthogonality and diversity, while also employing rational design.

### In vitro validation of peptide hits with GFP-peptide tags

Hit peptides were chosen after normalization (Supplementary Methods 4A–B) of fluorescence intensities on the SPOT array membrane after treatment with each of the two PPTases. Peptides that displayed orthogonal characteristics were selected as hits. These hits exhibited a high fluorescence intensity when labeled by one PPTase and low intensity when treated with the other.

**Fig. 5:**
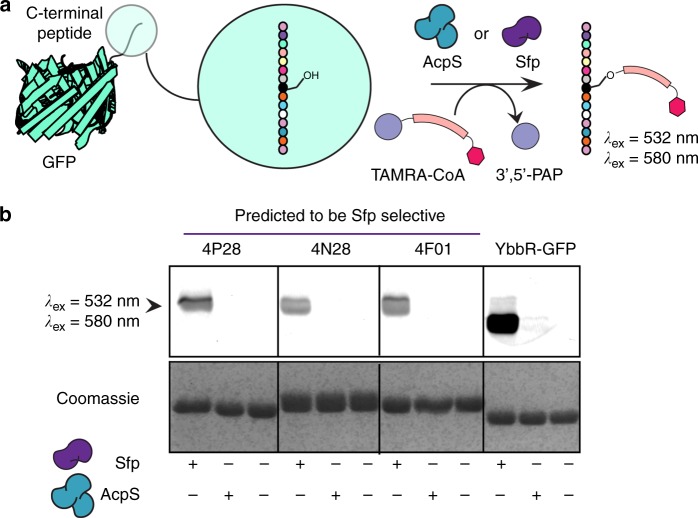
Validation of PPTase-specific peptide hits using GFP-peptide tags. **a** Modification of GFP-peptide fusions by addition of TAMRA-CoA onto selective peptides appended on the C*-*terminus of GFP. **b** Twelve percent SDS-PAGE gel corresponding to labeling (*λ*_ex_ =532 nm/ *λ*_em_ =580 nm) of GFP peptide tag by Sfp and AcpS. PPTase (Sfp and AcpS) is not observed due to low abundance (Coomassie sensitivity). TAMRA-CoA was present in each enzymatic reaction

Previous studies demonstrated that YbbR can be genetically encoded at the N- and C-termini of enhanced green fluorescent protein^24^. We sought to validate our hit peptide substrates identified on the membrane in order to confirm that no labeling artifacts were chosen as hits from the SPOT cellulose membrane. Hit peptides were encoded at the C-terminal end of super-folded GFP^29^. Recombinant purified GFP peptide tags were incubated individually with both Sfp and AcpS and TAMRA-CoA. As predicted by our POOL program, the Sfp-specific GFP -peptides were only labeled by Sfp, and not by AcpS (Fig. 5) (Supplementary Figure 4).

We found that we were unable to label our AcpS-type GFP peptides with our TAMRA-CoA analog after purification from *E. coli*. We hypothesized that these GFP peptides were modified by endogenous CoA and AcpS in *E. coli*. To test whether our AcpS-type GFP peptides were labeled by *E. coli* AcpS, in cellulo labeling with our previously published coumarin phosphopantetheine analog^27^ was confirmed by LCMS. Ions characteristic of coumarin 4′-phosphopantetheine were observed (Supplementary Figure 5)^30,31^.

To evaluate the catalytic efficiency of the Sfp-type GFP peptides, a kinetic analysis was conducted (Supplementary Table 1). The catalytic efficiency for labeling the various GFP peptide substrates was comparable to that of the previously published YbbR-GFP^24^.

Unlike previous phage display studies that identified similar peptide sequences, POOL was able to specifically hone in on important residues adjacent to the catalytic serine that increased the diversity of peptides recognized by Sfp and AcpS. POOL was trained to pick hit peptides, not necessarily catalytically efficient peptides. Future studies include incorporating kinetic data into the algorithm to be employed by an optimization strategy in subsequent rounds of POOL.

### Demonstrating orthogonal peptides on a membrane support

**Fig. 6:**
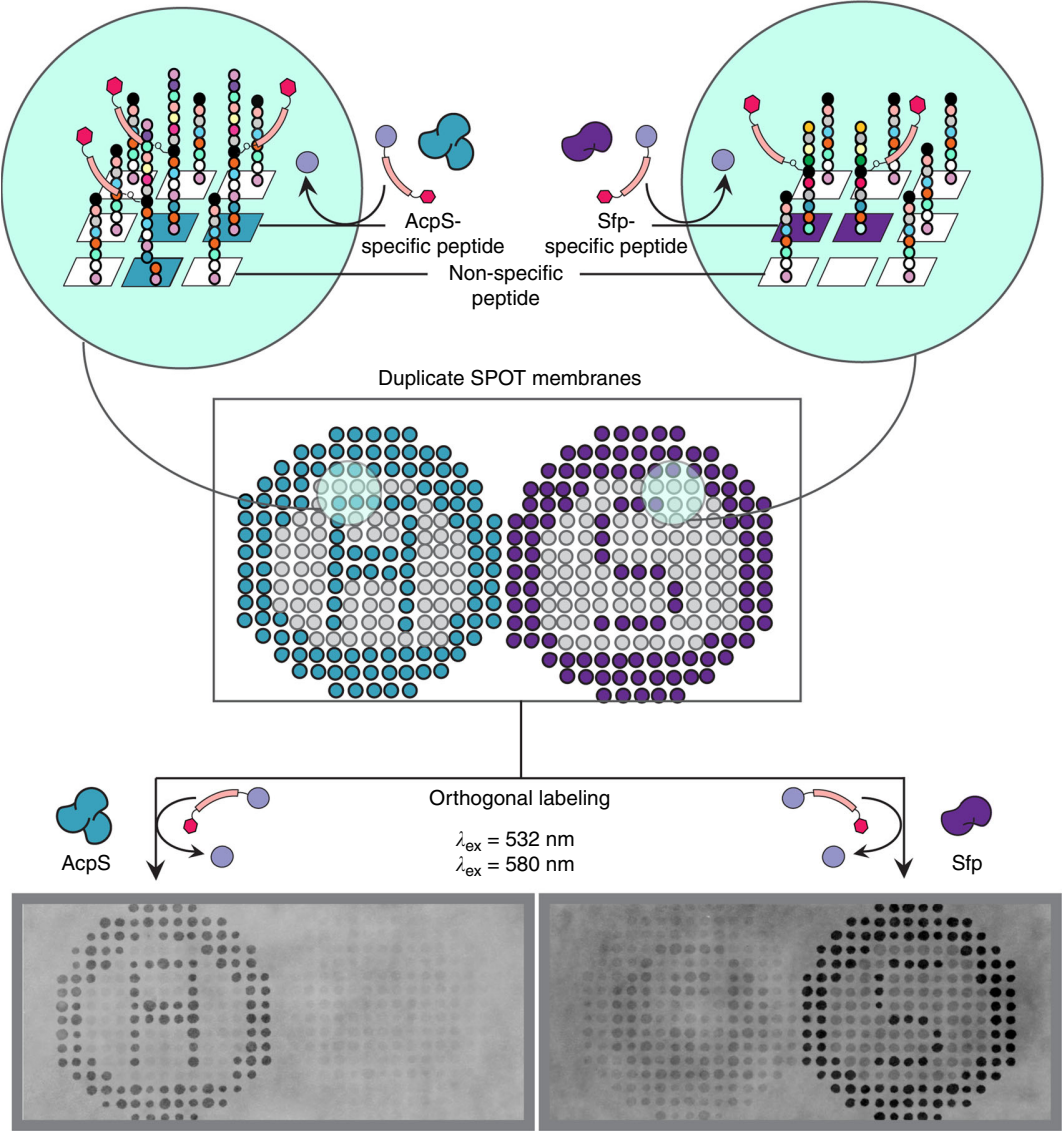
Scheme illustrating selective Sfp and AcpS labeling on duplicate SPOT membranes. Peptide AVKMESLEYLDTM (4F01), specific to Sfp, was synthesized on the cellulose membrane to highlight “S,” while peptide WPEEGIESFMSVPPP (4T25), specific to AcpS, highlighting “A.” All unfilled areas inside the circle were synthesized with a non-specific peptide, IHDGADSVVWLWSNC, which was neither a substrate for Sfp nor AcpS

To demonstrate peptide specificity for each of the two PPTases, the top lead peptide for each PPTase was synthesized on the surface of two duplicate cellulose membranes as two letters, “A” and “S.” The circle on the left-hand side (A) is specific to AcpS labeling, while the circle on the right-hand side (S) is specific to Sfp type (Fig. 6). The peptide AVKMESLEYLDTM (4F01), specific to Sfp, was synthesized on the cellulose membrane to highlight “S” (imaged on the right). Conversely, peptide WPEEGIESFMSVPPP (4T25), specific to AcpS, was synthesized on the cellulose membrane highlighting “A” (imaged on the left). All unfilled areas inside the circle were prepared with IHDGADSVVWLWSNC peptide, a sequence that is neither a substrate for Sfp nor AcpS. Duplicate membranes synthesized with the orthogonal and unspecific peptides were treated with TAMRA-CoA and either AcpS or Sfp. After treatment by AcpS with TAMRA-CoA, the peptides forming an “A” pattern were illuminated, while the duplicate membrane treated with Sfp illuminated the “S” peptides (Fig. 6). In all treatments, the non-specific peptides synthesized in all unfilled areas within the circles were neither labeled by Sfp nor AcpS as expected. These results demonstrate the selectivity for each of these enzyme isoforms for the newly discovered peptides via POOL.

## Discussion

Here, we demonstrate POOL’s rapid convergence on biomolecules with a desired activity. Unlike classical selection-based approaches, such as phage display and mutagenesis, POOL is efficient at simultaneously (1) searching for orthogonal activity; (2) exploring diverse peptide scaffolds beyond known substrates; (3) and judiciously selecting small sets of peptides rather than screening large, randomly generated libraries. Coupled with the SPOT technology, POOL offers a mathematically driven approach for discovery of structurally diverse orthogonal peptide substrates that are selective for multiple enzymes catalyzing the same enzymatic reaction.

POOL enabled the discovery of short, selective oligopeptide substrates for Sfp and AcpS. While POOL was utilized herein for PPTases and the identification of peptide substrates for these enzymes, our approach is generalizable and can be applied for the discovery of peptide substrates of other enzymes, such as kinases, proteases, and glycosyltransferases, which are therapeutically relevant enzyme targets^32–34^. A key advantage of POOL over traditional peptide substrate discovery methods is the ability to screen for multiple biological properties simultaneously. Here, we demonstrated the discovery of orthogonal peptides that are specific for different classes of PPTases. Furthermore, the orthogonal peptides differ significantly from each other and from their initial training set (Fig. 3c, d), which includes natural substrates^26^. That is, when compared to the mutation and predict-then-optimize methods, POOL enables an enhanced departure in peptide sequence space from the nearest training set, underscoring POOL’s ability to generate a wide diversity of evolved peptide substrates. Previously discovered orthogonal peptides maintained high sequence conservation toward the N-terminus when compared to the natural CP substrate and a constant length of 12 amino acids^26^. The orthogonal peptides found in POOL are beyond the initial peptide training set and differ in length (8–20 residues), position of the catalytic serine residue, and chemistry of residues surrounding the catalytic serine.

POOL is a powerful method that can be readily extended to a broad class of biochemical applications because the underlying machine learning model is a flexible classifier that can predict other properties from training data. By defining a hit as any biochemical property or outcome of interest in parallel with experimental data, POOL’s predictive model can be trained to guide the discovery process towards any application. Additionally, POOL is a flexible method that can be adapted beyond the naive Bayes prediction model to a wide range of complementary machine learning models, while still maintaining its theoretically guaranteed performance (Supplementary Methods 2E). We envision applying and adapting POOL to identify biologically active peptide substrates important for epitope mapping, antigen discovery, receptors, and natural ligands that are therapeutically relevant. Additionally, POOL can be applied toward identifying peptide substrates important for characterizing enzymatic functions, while simultaneously screening for multiple biochemical activities.

## Methods

### Bayesian machine learning and POOL process

The prediction model used in POOL is a Bayesian variant of naive Bayes, modified to incorporate the belief that amino acids far from the central serine at which modification occurs have less impact on activity. Despite the word naive in its name, naive Bayes is a simple but powerful classification method widely used within machine learning for text classification. POOL then selects peptides to add iteratively by maximizing the conditional probability of being a hit, conditioned on previously added peptides in the batch not being hits. It performs this maximization across all peptides with a target length. Details of the method are provided in Supplementary Methods 1A.

### Preparation of super-folded GFP peptide constructs

Super-folded GFP^29^ gBlock gene fragment (IDT) was subcloned into the *Nde*I–*Xho*I site of a pET28b vector (Novagen) containing an N-terminal His6-tag. The Sfp-type (4P28, 4N28, and 4F01) and AcpS-type (1F01, 1I04, 3K17, and 4T25) peptides for the various GFP constructs were ordered as single-stranded oligonucleotides (forward and reverse) with 5′-phosphorylation (IDT) and its corresponding *Xho*I overhangs. To prepare the double-stranded oligonucleotides, the forward and reverse single-stranded oligonucleotides were mixed together in equal molar amounts in 100 mM potassium acetate; 30 mM HEPES, pH 7.5. The equimolar forward and reverse oligos were heated to 94 °C for 2 min and slowly cooled by decreasing the temperature 1 °C every 30 s to a final temperature of 4 °C. The double-stranded oligomer was ligated into the corresponding *Xho*I (NEB) cut vector. These plasmids were transformed into an *E. coli* BL21 Δ*entD* strain, which was a gift from Professor D.F. Ackerley^35^.

### Recombinant protein purification

PPTases, *B. subtilis* Sfp, *S. coelicolor* AcpS, and GFP-peptide proteins were expressed and purified^29,36^. Cells were grown at 37 °C in terrific broth media containing 100 mg L^−1^ kanamycin sulfate to an optical density at 600 nm (OD_600_) of 0.8. The cells were induced with a final concentration of 250 mM of isopropyl-β-d-thiogalactopyranoside (IPTG) and grown overnight at 16 °C. The cells were harvested by centrifugation at 2000 × *g* for 30 min, resuspended in lysis buffer (50 mM Tris-HCl buffer, pH 7.5, with 250 mM NaCl), and supplemented with 0.1 mg mL^−1^ lysozyme (Worthington Biochemical Corp), 5 µg mL^−1^ DNAse I (Sigma), and 5 µg mL^−1^ RNAse (Worthington Biochemical Corp.). The cells were lysed by a French pressure cell press between 500 and 1000 psi. The lysate was subsequently centrifuged at 12,000 × *g* for 45 min, and the supernatant was bound with Ni-NTA resin (Qiagen). The column was sequentially washed with the wash buffer (50 mM Tris-HCl buffer, pH 7.5, 250 mM NaCl, 25% glycerol, 10 mM buffered imidazole), followed by elution at 300 mM buffered imidazole. The purified protein was desalted into 50 mM Tris-HCl buffer, pH 7.5, with a PD-10 desalting column (GE Healthcare Life Sciences) and concentrated with a 10-kDa Amicon spin filter (Millipore Sigma). The concentrated sample was subsequently stored in 20% glycerol, 50 mM Tris-HCl buffer, pH 7.4, with 150 mM NaCl at −80 °C after flash freezing in liquid nitrogen.

### SPOT synthesis of cellulose membrane peptides

The peptides were synthesized on an amino-PEG-cellulose 10×15 cm^2^ membrane and synthesized automatically by MultiPep RSi (INTAVIS Bioanalytical Instruments AG). After Fmoc cleavage with 20% 4-methyl piperidine (Sigma) in dimethylformamide (DMF), activated with 1 equiv. hydroxybenzotriazole and 2 equiv. *N*-methylpyrrolidone (NMP), and directly spotted on the membrane; after 15 min this step was repeated. The membrane was washed with DMF (3 × 3 min). Solutions of the Fmoc amino acids in NMP were spotted on the membrane (0.6 M solutions; 0.8 M solutions for C, H, N, Q, and R; triple coupling, 15 min each). The Fmoc group was removed from the spots, and the sequences of the peptides were completed using the standard SPOT synthesis protocol and followed by an N-terminal tag. Membranes were stored at −20 °C until treated.

### Membrane treatment

Synthesized membranes were deprotected with cleavage cocktail consisting of 94% trifluoroacetic acid (TFA), 2.5% water, 1% triisopropylsilane (Sigma), and 2.5% 1,2-ethanedithiol (Sigma) shaking at 2 h at room temperature. The membranes were washed in triplicate with dichloromethane (Sigma), ethanol (Sigma), distilled deionized water, and Tween-20 Tris-buffered saline (TBST) (20 mM Tris-HCl buffer, pH 7.4, with 150 mM NaCl), respectively. The membrane was incubated with 5% bovine serum albumin (BSA) in TBST for 1 h at room temperature. Following blocking by BSA, the membranes were washed three times with TBST. The membranes were subjected to labeling by their respective PPTase, either *B. subtilis* Sfp or *S. coelicolor* AcpS. The final 20 mL reaction volume consisted of 10 µM PPTase enzyme, 40 µM TAMRA-CoA, 0.01% Triton X, and 15 µM BSA in 50 mM Tris-HCl buffer, pH 8.0, and 10 mM MgCl_2_. The reaction was incubated for 16 h at 37 °C. The membranes were rinsed liberally with TBST buffer, 0.1% TFA, and distilled deionized water before imaging on a Typhoon TRIO Variable Mode Imager (GE Healthcare BioSciences) at 50-µm resolution with 532 nm green laser excitation and 580 nm emission filter and a photomultiplier tube setting of 350 V. The membrane images were analyzed by the ImageJ software^37,38^.

### In vitro validation of peptide hits with GFP-peptide tags

Three Sfp-type (4P28, 4N28, and 4F01) and four AcpS-type (1F01, 1I04, 3K17, and 4T25) peptides were fused to the C*-*terminal end of super-folded GFP^29^. To access orthogonal labeling, these specific GFP-peptide tags were incubated with either Sfp or AcpS in the presence of TAMRA-CoA (4). The reactions were conducted in a 30 µL reaction containing a final concentration of 10 µM of the respective GFP-peptide tag, 5 µM TAMRA-CoA (4), and 0.05 µM of either Sfp or AcpS in 50 mM HEPES, 10 mM MgCl_2_, pH 7.2. The mixture was gently shaken at 37 °C overnight. The resulting reactions were analyzed by 12% sodium dodecyl sulfate-polyacrylamide gel electrophoresis (SDS-PAGE) gel and imaged on a Typhoon TRIO Variable Mode Imager (GE Healthcare BioSciences) at 50-µm resolution with 532 nm green laser excitation and 580 nm emission filter and a photomultiplier tube setting of 350 V.

### PPTase gel-based electrophoretic mobility shift kinetics

A concentration-dependent assay varying concentrations of GFP peptides was conducted to characterize the kinetics of all the GFP peptides with Sfp^39^. Briefly, reaction was initiated by the addition of 0.1 µM Sfp into a 30 µL reaction containing 1 mM TAMRA-CoA buffer (50 mM HEPES, 10 mM MgCl_2_, pH 7.2), and with varying concentrations of GFP peptide (0.1– 10 µM). The reactions were quenched every 5 min sequentially by adding 100 mM EDTA. Resulting reactions were analyzed by 12% SDS-PAGE gel and imaged from Typhoon (GE Healthcare) scanner (λ_ex_ = 532 nm/λ_em_ **=** 580 nm). The intensity of the fluorescence gel bands were measured and analyzed using GraphPad Prism and fitted to the Michaelis–Menten equation, *Y* = *V*_max_ × *X/*(*K*_m_ + *X*), where *K*_m_ is the Michaelis–Menten constant, *X* is the substrate concentration, *V*_max_ is the maximum enzyme velocity, and *Y* is the velocity of the enzyme. The data were collected in triplicate (Supplementary Table 1).

### In cellulo labeling and LC-MS analysis of AcpS GFP peptides

GFP peptides encoded in a pET28b plasmid in *E. coli* BL21 (Δ*entD*) cells were grown for 5 h at 37 °C in 5 mL LB medium containing 100 mg L^−1^ kanamycin sulfate to an OD_600_ of 0.6–0.8 and induced with a final concentration of 500 µM IPTG. Along with the addition of IPTG, 1 mM of coumarin phosphopantetheine analog was added into the growth medium to investigate uptake and labeling^27^. The cultures were grown for 16 h at 16 °C. The cells were centrifuged and resuspended in lysis buffer (50 mM sodium phosphate, 300 mM NaCl, pH 7.5), and lysed by incubation with lysozyme (1 mg ml^−1^) for 1 h on ice followed by sonication (3 × 10 second pulses) and centrifugation. 50 µL of protein lysate of GFP peptide at a concentration of 5 µM was precipitated by the addition of 1 mL trichloroacetic acid solution (10% weight per volume percent) and pelleted by centrifugation at 4 °C at 14,000 rpm for 20 min. The pellet was washed with 1 mL water twice and centrifuged at 4 °C at 14,000 rpm for 20 min after each washing step. Subsequently, 100 µL of 0.1 M KOH was added and the sample was heated for 30 min at 75 °C. Then, the protein was precipitated with 10 µL trifluoroacetic acid (50% volume per volume percent) followed by centrifugation as before, and the supernatant was analyzed by LC-MS (liquid chromatography-mass spectrometry) under single-ion detection mode. Hydrolyzed coumarin phosphopantetheine (*m*/*z* = 626.64) was detected^30^.

### Sequence alignment

Sequence alignment was conducted using the open online software ESPript 3.0^40^.

### Code availability

Code is available at https://github.com/peter-i-frazier/pool. Code is published under an Apache 2.0 license.

## Electronic supplementary material

Supplementary Information

## Data Availability

The data are freely available at https://github.com/peter-i-frazier/pool.
