# Supplementary material for: Discovering de novo peptide substrates for enzymes using machine learning
Source: Nat Commun. 2018 Dec 7;9:5253. doi: 10.1038/s41467-018-07717-6 (PMC6286390; doi:10.1038/s41467-018-07717-6)
Supplement: Supplementary file 1 — Supplementary Information [file 41467_2018_7717_MOESM1_ESM.pdf]

Supporting Information
Discovering *de novo* peptide substrates for enzymes using machine learning

Tallorin et al.

Supplementary Methods

1. Use of POOL methodology for discovery of peptides for orthogonal labeling

The POOL model was initially trained by three categories of peptides to generate short orthogonal active peptide substrates. First, we obtained peptides by truncating portions of acyl carrier proteins from various organisms known to be substrates for the following enzymes: *B.subtilis* Sfp [1], *S. coelicolor* AcpS [2], and *P. fluorescens* AcpH [3]. (AcpH is an enzyme known to unlabel some substrates previously labeled by a PPTase, either AcpS or Sfp.) Second, we included short peptide substrates that were previously [1, 2] identified as substrates for PPTases. Third, we included truncated acyl carrier proteins from various organisms and short peptides known not be substrates (inactive) for the previous three enzymes [3]. Next, the POOL algorithm interactively queried the experimentalists to test a group of desired peptides (usually 300-500 peptides) to identify active short hits. We incorporated the test results as new data points into the dataset to update the learning model. We repeated this process iteratively for 5 rounds.

In each round, the POOL algorithm is told the type or types of hits to discover, and it allocates the available membrane space among them. For example, in one round, POOL may be told to search for both Sfp hits (peptides that are active for Sfp, regardless of their activity with AcpS and AcpH) and AcpS hits (peptides that are active for AcpS), in which case it would choose an equal number of peptides to test for each goal. Other types of hits to which POOL may be applied include Sfp-specific hits (active for Sfp and not active for AcpS), AcpS-specific hits (active for AcpS and not active for Sfp), Sfp-specific hits with unlabeling (active for Sfp and AcpH and not active for AcpS), and AcpS-specific hits with unlabeling (active for AcpS and AcpH and not active for Sfp).

In the first and second rounds, POOL searched for Sfp hits with unlabeling, while in rounds three through five POOL searched for Sfp-specific hits with unlabeling and AcpS-specific hits with unlabeling. In the first and second rounds, Sfp labeling and AcpH unlabeling activity was measured. Then at the start of round three, AcpS labeling and AcpH unlabeling activity was measured for the peptides tested in round 1. Sfp labeling with AcpH unlabeling, and AcpS labeling with AcpH unlabeling were tested for rounds 3 through 5. Thus, measurements required for assessing specific labeling were conducted for four rounds in total.

We originally had the goal of discovering substrates that were labeled by a PPTase and unlabeled by AcpH, optionally with specific activity for one PPTase, and directed POOL toward this goal. Later, we chose to focus on the specific labeling portion of this goal, without the extra complication of unlabeling. While ongoing studies in our laboratories are looking at subsequent processing steps, adding the extra requirement that modifcations could be removed by hydrolases, AcpH unlabeling is not relevant for this manuscript.

As discussed in the main text and in more detail in the next section, the POOL algorithm comprises two pieces (see Figure 1). The first uses a machine learning method, Naive Bayes [4], to predict the probability that a given peptide sequence will be a hit based upon previously collected training data. The second uses value of information analysis [5, 6], together with the probabilities calculated by Naive Bayes, to find a set of peptides to test next that, considered together as a group, have maximum probability of containing at least one short hit. Both simulations and an analysis of previously obtained experimental data demonstrate that using POOL dramatically reduces the number of experiments required to find short hits, as compared with a baseline method of randomly mutating and truncating previously known substrates.

2. Description of the POOL methodology

We first introduce mathematical notation that allows a more precise discussion of the POOL methodology. Let E be the set of peptides over which we would like to search, which we take be the set of peptides of length less than 38 amino acids. For any peptide sequence $e \in E$, let $y(e) \in \{0, 1\}$ be a binary label indicating whether peptide e is a hit, where the definition of hit is specific to the goal at hand (e.g., discovering Sfp hits, or Sfp-specific hits). This label can be observed directly through experiment, but is otherwise unknown *a priori*.

We let $f(e)$ be equal to negative one times the length of the peptide. The negative one is present because we will want $f(e)$ to be large, but we want the length to be small. We will sometimes refer to $f(e)$ generically as the "fitness".

Our goal, in terms of the notation we have defined, is to find a peptide $e \in E$ for which $y(e) = 1$ and for which $f(e)$ is as large as possible. We can write this problem as,

$$\underset{e \in E, y(e)=1}{\arg\max} \; f(e). \qquad [1]$$

POOL chooses a set of peptides S to test in a single round, to best support solving Eq. (1), in the specific sense of maximizing the probability of finding a hit whose fitness is better than some target fitness b. It consists of three steps: first, a prediction step, in which a machine learning method (Naive Bayes, customized for peptide activity) provides a joint probability distribution over peptide activity; second, a probability-of-improvement step, in which we use value-of-information analysis to define the quality of a set of peptides to test; and third, a greedy optimization step, in which we use a greedy algorithm to find a set of peptides to test that provides a large probability of improvement.

Below, Section A describes the machine learning model POOL uses to predict $y(e)$; Section C defines the probability of improvement criterion that POOL seeks to maximize in the set of peptides it recommends testing; Section D,F and G describes the greedy approach that POOL uses to approximately maximize the probability of improvement; Section E provides a theoretical guarantee on the solution quality from this greedy approach; and Section H discusses how POOL can be used when only partial labeling information is available for some peptides in the training set.

A. Bayesian Machine Learning Model. POOL's prediction step uses a modified Naive Bayes classifier within a Bayesian framework to provide prediction of a joint probability distribution over peptide activity. Naive Bayes classifiers [7, 8] are popular and extensively studied in text classification problems because of their fast computation and ease of implementation. As peptides are sequences of amino acids just like documents are sequences of words, peptide classification is similar to text classification, and similar methods can be used. Rather than using the classical Naive Bayes directly, we modify it by putting a Bayesian prior distribution on the model's parameters. This supports higher-quality inference with small amounts of data.

We now explain the approach in detail.

Since the chemoenzymatic reaction happens at the conserved Serine, we are looking for the peptides containing exactly one Serine in the sequence. We use Serine as the origin, and label positions of other amino acids in the sequence by their sequence-distance from the origin in the *C*-terminus or *N*-terminus direction. Each position contains one feature, which is determined by the amino acid residing in this position. To reduce dimensionality of the feature space, we group the 20 standard amino acids into 8 classes (shown in Supplementary Figure 2) according to chemical type, and represent the amino acid by its class. For example, a sequence X-X-X-S-X-X, where S represents Serine and X represents the reduced amino acid alphabet, can be written in this feature representation as N3-N2-N1-S-C1-C2, where N# and C# are the groups of the amino acids in the corresponding position.

We use a J-dimensional feature vector to represent a peptide, written as $\mathbf{x} = (x_1, \ldots, x_J)$, and the feature space is $\mathcal{X}$. In our application, each feature corresponds to a position in the peptide, and the feature variable indicates which the amino acid class is present at that position. Then we can predict $y(e)$ by calculating $\mathbb{P}(y(e) = 1 \mid \mathbf{x})$. Using Bayes' theorem, we have

$$\mathbb{P}(y(e) = 1 \mid \mathbf{x}) = \frac{\mathbb{P}(\mathbf{x} \mid y(e) = 1)\, \mathbb{P}(y(e) = 1)}{\sum_{y' \in \{0,1\}} \mathbb{P}(\mathbf{x} \mid y(e) = y')\, \mathbb{P}(y(e) = y')}. \quad [2]$$

Since Naive Bayes assumes the features are conditionally independent given y', we can write Eq. (2) as

$$\mathbb{P}(y(e) = 1 \mid \mathbf{x}) = \frac{\prod_{j=1}^{J} \mathbb{P}(x_j \mid y(e) = 1)\, \mathbb{P}(y(e) = 1)}{\sum_{y'=0}^{1} \prod_{j=1}^{J} \mathbb{P}(x_j \mid y(e) = y')\, \mathbb{P}(y(e) = y')}. \quad [3]$$

If we knew $\mathbb{P}(x_j \mid y(e) = y')$ and $\mathbb{P}(y(e) = y')$ for $y' \in \{0, 1\}$, we could use Eq. (3) to compute $\mathbb{P}(y(e) = 1 \mid \mathbf{x})$. Our next task is to estimate both quantities from data.

We will adopt a Bayesian viewpoint in performing this estimation. Toward this end, we augment our model by adding a set of unknown latent random variables $\theta_i^{y',j}$ where j ranges across positions, $y' \in 0, 1$, and i ranges across the K distinct groups of amino acids available for selection at position j. $\theta_i^{y',j}$ will represent the frequency with which an amino acid in class j arises at position i among peptides with label y. We let $\boldsymbol{\theta}^{y',j} = (\theta_1^{y',j}, \ldots, \theta_K^{y',j})$ and $\boldsymbol{\theta}$ be the matrix comprised of $\boldsymbol{\theta}^{y',j}$ with $y' \in \{0, 1\}$ and j across the set of positions. In this Bayesian context, Eq. (2) and Eq. (3) become

$$\mathbb{P}(y(e) = 1 \mid \mathbf{x}, \boldsymbol{\theta}) = \frac{\prod_{j=1}^{J} \mathbb{P}(x_j \mid y(e) = 1, \boldsymbol{\theta})\, \mathbb{P}(y(e) = 1 \mid \boldsymbol{\theta})}{\sum_{y'=0}^{1} \prod_{j=1}^{J} \mathbb{P}(x_j \mid y(e) = y', \boldsymbol{\theta})\, \mathbb{P}(y(e) = y' \mid \boldsymbol{\theta})}. \quad [4]$$

We then define $\mathbb{P}(x_j \mid y(e) = y', \boldsymbol{\theta}) = \theta_{x_j}^{y',j}$ and $\mathbb{P}(y(e) = 1 \mid \boldsymbol{\theta}) = \mathbb{P}(y(e) = 1)$.

We further put a Dirichlet prior over $\boldsymbol{\theta}^{y',j}$, that is, $\boldsymbol{\theta}^{y',j} \sim \text{Dirichlet}(\boldsymbol{\alpha}^{y,j})$. We set $\boldsymbol{\alpha}^{y,j}$ to be a vector that grows with the square root as we move further from the conversed serine: $\alpha_i^{y,j} = \alpha_i^{y,1}\sqrt{|j|}$. A Dirichlet prior on $\boldsymbol{\theta}^{y,j}$ becomes more concentrated around its mean $\boldsymbol{\alpha}^{y,j} / \sum_i \alpha_i^{y,j}$ as we increase $\sum_i \alpha_i^{y,j}$, and so this causes us to put more weight in our prior on the belief that $\boldsymbol{\theta}^{y,j}$ is close to $\boldsymbol{\alpha}^{y,1} / \sum_i \alpha_i^{y,1}$ as we move away from the conversed serine. We choose $\boldsymbol{\alpha}^{y,1}$ so that $\boldsymbol{\alpha}^{y,1} / \sum_i \alpha_i^{y,1}$ is identical for $y = 0$ and $y = 1$, and so as we move away from the serine we become more confident that $\boldsymbol{\theta}^{y,j}$ is similar across y, which corresponds to this position having less influence on whether the peptide is active or not.

If $\alpha_i^{y,1}$ is proportional to number of amino acids in class i, then the mean value $\boldsymbol{\alpha}^{y,1} / \sum_i \alpha_i^{y,1}$ corresponds to the amino acid class proportions we would expect at a position that has no influence on activity. Also, it would be appropriate to set the magnitude of $\sum_i \alpha_i^{y,1}$ to be larger for $y = 0$ than for $y = 1$, since most peptides are inactive causing the distribution over amino acid classes among inactive peptides to more closely mimic this distribution at all positions. We realized the importance of class size in this analysis after conducting experiments: $\boldsymbol{\alpha}^{y,1}$ used to recommend peptides in our experiments was instead proportional to the unit vector, with a larger proportionality constant for $y = 1$.

With this Dirichlet prior, the posterior distribution of $\boldsymbol{\theta}^{y',j}$ given training data $\{(\mathbf{x}^1, y^1), \ldots, (\mathbf{x}^N, y^N)\}$ is also a Dirichlet distribution, with updated parameters $\tilde{\alpha}_k^{y',j} = \alpha^{y',j} + \sum_{n=1}^{N} \mathbb{1}_{\{y^n = y', x_j^n = k\}}$, for $k = \{1, \ldots, K\}$.

To estimate the conditional probability that $y(e) = 1$ given data, we sample $\boldsymbol{\theta}$ from its posterior distribution for all y and j, calculate the conditional probability that $y(e) = 1$ given $\boldsymbol{\theta}$ using Eq. (4), and then average across samples. We can calculate a joint conditional distribution on $y(e)$ for a set of e given data using a similar technique.

B. Accuracy of the Bayesian Machine Learning Model. While Figure 2 C-D show that the predictions from POOL's prediction step are substantially better than random chance, they are imperfect. This imperfection is due to four factors.

First, the reduced amino acid alphabet does not distinguish between amino acids in the same class.

Second, Naive Bayes assumes conditional independence, but instead it may be that an amino acid at one position is more effective at generating activity when an adjacent position has a particular amino acid present.

Third, because most of the training data used in prediction was generated by POOL's past recommendations, it consists largely of peptides that were likely to be a hit according to the predictive model at the time they were selected. However, hits are rare, and so only a fraction of these are actually hits. Moreover, this generation process causes more of the peptides in the training data to have similar representations in feature space than if the data had been generated uniformly at random. These characteristics cause the prediction problem with results pictured in Figure 2 C-D to be particularly difficult. In contrast, if the training data had been generated at random, the misses would be better separated from the hits in the model's feature space, and it would be easier for POOL's predictive model to provide accurate classification.

Fourth, the function POOL's predictive model is trying to learn (the mapping from peptides onto activity) has a number of degrees of freedom substantially larger than the amount of training data available. With only the 10 amino acids closest to the Serine residue one may create 20^{10} (approximately 10^{13}) peptides. The mapping from peptides onto activity would predict, for each of these peptides, what its activity is. Representing an arbitrary function of this form requires $2^{20^{10}}$ bits. This is many orders of magnitude larger than the amount of information present in POOL's training data (activity for 2600 peptides, considering all rounds).

While one could address the first and second issues above through a more complex model with more parameters, the predictive model POOL uses already contains 2*(8-1)=14 parameters at each location (the prevalence of each class in the populations of hits and misses respectively, minus 1 for the need for these prevalence to sum to 1), giving 14*20=280 parameters to estimate over 20 locations. Moreover, POOL must estimate these parameters while avoiding overfitting not just after the final round, but before the start of each round. At the start first round, there were only 40 peptides in our training data, and at the start of the second round only 500. For this reason, not enough data is present to allow accurate estimation of the additional parameters in a substantially more flexible model.

Thus, POOL uses a reduced amino acid alphabet and assumes conditional independence because the reduction in the number of parameters required offers substantial advantages when making predictions with relatively little data. While a more complex model could have better predictive accuracy after substantially more data was collected, it would be likely to have worse accuracy in the data-poor regimes where peptide discovery typically operates.

C. Probability of improvement. POOL uses the joint probability distribution over $y(e) : e \in E$ provided by Naive Bayes to define an auxiliary function, called probability of improvement, which quantifies the probability that a set of peptides S, if tested, will reveal an active peptide whose fitness $f(e)$ improves on some benchmark value b, which is typically the best $f(e)$ of active peptides observed in the past. We define this probability-of-improvement, $\mathrm{PI}(S)$, to be

$$\mathrm{PI}(S) = \mathbb{P}\left(\max_{e \in S, y(e)=1} f(e) > b\right). \qquad [5]$$

In the event that all peptides tested are inactive, we define max over an empty set to be $-\infty$. POOL seeks to set S by approximately solving

$$\arg\max_{S \subseteq E, |S| \leq k} \mathrm{PI}(S), \qquad [6]$$

where k is the number of peptides that can be tested simultaneously in a single round of experiment.

After we find the set S and tested the peptides in it, we use the results along with previously tested peptides as training data, and find a new set S for the next round of experiment. We repeat this process until we find enough favored active peptides or resource is exhausted.

D. Maximizing probability of improvement using the greedy algorithm. Directly solving Eq. (6) is computationally challenging, first because the cost of computing $\mathrm{PI}(S)$ grows exponentially in k, and second because the number of sets S satisfying $|S| \leq k$ also grows exponentially in k. In this section we describe an easily computed approximate solution to Eq. (6). The method we employ is a greedy algorithm, which starts with $S = \emptyset$, and then adds peptides one-by-one, each time choosing the one that will increase the probability of improvement by the greatest amount, that is, solving

$$\arg\max_{e \in E \setminus S} \mathrm{PI}(S \cup \{e\}), \qquad [7]$$

until $|S| = k$. A theoretical result provided in E provides a performance guarantee on solution quality in the form of a lower bound on the ratio between the probability of improvement provided by the greedy approach and solving Eq. (5) exactly.

E. Performance guarantee for the greedy algorithm. The following theorem provides a guarantee on the probability of improvement provided by the greedy algorithm relative to that provided by solving Eq. (6) exactly.

Theorem 1. The greedy algorithm always produces a solution whose probability-of-improvement is at least $1-[(k-1)/k]^k \geq 1-1/\mathrm{e}$ times the optimal objective value of Eq. (6).

The proof of the theorem follows directly from the following two lemmas. The first lemma is shown in [9] while we provide a proof of the second.

Lemma 1. If $F(S)$ is submodular, non-decreasing and $F(\emptyset) = 0$, the greedy heuristic always produces a solution whose value is at least $1 - [(k-1)/k]^k$ times the optimal value, where $|S| \leq k$. This bound can be achieved for each k and has a limiting value of $1 - 1/\mathrm{e}$, where e is the base of the natural logarithm.

Lemma 2. $\mathrm{PI}(S)$ is submodular, non-decreasing and $\mathrm{PI}(\emptyset) = 0$.

Proof. First we show $\mathrm{PI}(\emptyset) = 0$.

$$\mathrm{PI}(\emptyset) = \mathbb{P}(f^*(\emptyset) > b) = \mathbb{P}(-\infty > b) = 0.$$

To show $\mathrm{PI}(S)$ is non-decreasing, let $A \subseteq B \subseteq E$ where E is a finite set. Then

$$\begin{aligned}
\mathrm{PI}(B) &= \mathbb{P}(f^*(B) > b) \\
&= \mathbb{P}(f^*(B) > b \mid f^*(A) \leq b)\mathbb{P}(f^*(A) \leq b) \\
&\quad + \mathbb{P}(f^*(B) > b \mid f^*(A) > b)\mathbb{P}(f^*(A) > b) \\
&= \mathbb{P}(f^*(B) > b \mid f^*(A) \leq b)\mathbb{P}(f^*(A) \leq b) + \mathbb{P}(f^*(A) > b) \\
&\geq \mathbb{P}(f^*(A) > b) \\
&= \mathrm{PI}(A).
\end{aligned}$$

Lastly, we show PI(S) is submodular. For $e \in E \backslash B$,

$$\begin{aligned}
&\mathrm{PI}(A \cup \{e\}) - \mathrm{PI}(A) \\
&= \mathbb{P}(f^*(A \cup \{e\}) > b) - \mathbb{P}(f^*(A) > b) \\
&= \mathbb{P}(f^*(A \cup \{e\}) > b \mid f^*(A) > b)\mathbb{P}(f^*(A) > b) \\
&\quad + \mathbb{P}(f^*(A \cup \{e\}) > b \mid f^*(A) \leq b)\mathbb{P}(f^*(A) \leq b) - \mathbb{P}(f^*(A) > b) \\
&= \mathbb{P}(f^*(A \cup \{e\}) > b \mid f^*(A) \leq b)\mathbb{P}(f^*(A) \leq b) \\
&= \mathbb{P}(f(e) > b, y(e) = 1 \mid f^*(A) \leq b)\mathbb{P}(f^*(A) \leq b) \\
&= \mathbb{P}(f(e) > b, y(e) = 1, f^*(A) \leq b).
\end{aligned}$$

Using a similar argument,

$$\begin{aligned}
&\mathrm{PI}(B \cup \{e\}) - \mathrm{PI}(B) \\
&= \mathbb{P}(f(e) > b, y(e) = 1, f^*(B) \leq b) \\
&= \mathbb{P}(f(e) > b, y(e) = 1, f^*(A) \leq b, f^*(B \backslash A) \leq b).
\end{aligned}$$

Therefore, $\mathrm{PI}(A \cup \{e\}) - \mathrm{PI}(A) \geq \mathrm{PI}(B \cup \{e\}) - \mathrm{PI}(B)$. Thus PI($S$) is submodular. □

F. Simplifying the greedy algorithm. Although Eq. (7) is easier to solve than Eq. (6), it is nevertheless computationally infeasible to solve Eq. (7) using a naive approach in which we simply enumerate all peptides $e \in E \backslash S$ and calculate $\mathrm{PI}(S \cup \{e\})$ for each. This naive approach is infeasible because E is very large and calculating $\mathrm{PI}(S \cup \{e\})$ can take significant computational effort. To overcome these challenges we first transform Eq. (7) into an easy-to-compute objective.

Proposition 1. The solution to Eq. (7) is equal to

$$\underset{e \in E \backslash S, f(e) > b}{\arg\max} \quad \mathbb{P}(y(e) = 1 \mid y(e') = 0, \forall e' \in S). \qquad [8]$$

Proof. Let $f^*(S) = \max_{e \in S, y(e)=1} f(e)$. Then,

$$\begin{aligned}
&\mathrm{PI}(S \cup \{e\}) = \mathbb{P}(f^*(S \cup \{e\}) > b) \\
&= \mathbb{P}(f^*(S) > b) + \mathbb{P}(f^*(S) \leq b)\mathbb{P}(f(e) > b, y(e) = 1 \mid f^*(S) \leq b).
\end{aligned}$$

Since $P(f^*(S) \leq b)$ is strictly positive and does not depend on e, Eq. (7) is equal to

$$\underset{e \in E \backslash S}{\arg\max}\, \mathbb{P}(f(e) > b, y(e) = 1 \mid f^*(S) \leq b). \qquad [9]$$

Moreover, when $f(e) \leq b$, $\mathbb{P}(f(e) > b, y(e) = 1 \mid f^*(S) \leq b) = 0$. Thus the solution to Eq. (9) is equal to

$$\underset{e \in E \backslash S, f(e) > b}{\arg\max} \quad \mathbb{P}(y(e) = 1 \mid y(e') = 0, \forall e' \in S).$$

□

G. Implementing the greedy algorithm. Although Eq. (8) avoids the challenge of computing the probability of improvement, it is nevertheless hard to solve directly through enumeration, because E is large. Here we provide an efficient approximate implementation. (While the performance guarantee we show in Supplementary Section E does not apply to this approximate implementation, it neveretheless suggests that this approximation should perform well, which is also supported by simulation experiments shown in the main paper in Figures 3A and 3B.)

This approximate implementation relies on approximating the Bayesian posterior on $\boldsymbol{\theta}$ from our Naive Bayes predictive model by a point estimator $\hat{\boldsymbol{\theta}}$ of $\boldsymbol{\theta}$, such as the maximum *a posteriori* (MAP) estimator [10], or the mean of the posterior distribution. With any such point estimator our Naive Bayes predictive model becomes

$$\begin{aligned}
&\mathbb{P}\left(y(e) = 1 \mid \mathbf{x}\right) \\
&\approx \frac{\prod_{j=1}^{J} \hat{\theta}_{x_j}^{1,j} \mathbb{P}\left(y(e) = 1\right)}{\prod_{j=1}^{J} \hat{\theta}_{x_j}^{1,j} \mathbb{P}\left(y(e) = 1\right) + \prod_{j=1}^{J} \hat{\theta}_{x_j}^{0,j} \mathbb{P}\left(y(e) = 0\right)}.
\end{aligned}$$

Now we can write Eq. (8) as

$$\max_{\mathbf{x}} \frac{\prod_{j=1}^{J} \hat{\eta}_{x_j}^{j}}{\prod_{j=1}^{J} \hat{\eta}_{x_j}^{j} + \frac{\mathbb{P}(y(e)=0)}{\mathbb{P}(y(e)=1)}}, \qquad [10]$$

where $\hat{\eta}_{x_j}^{j} = \frac{\hat{\theta}_{x_j}^{1,j}}{\hat{\theta}_{x_j}^{0,j}}$. Since $\hat{\eta}_i^j \geq 0$, the objective in Eq. (10) is monotone increasing with $\hat{\eta}_{x_j}^{j}, \forall j$. Thus, we can solve Eq. (10) by maximizing $\prod_{j=1}^{J} \hat{\eta}_{x_j}^{j}$ over $\mathbf{x}$. Since $\hat{\eta}_{x_j}^{j}$ are independent across j in the Naive Bayes model, we can decompose the objective across j and maximize $\hat{\eta}_{x_j}^{j}$ by setting $x_j = \arg\max_i \hat{\eta}_i^j, \forall j$. The property of decomposition across j in this formulation greatly simplifies the optimization problem allowing an extremely efficient implementation.

This can be done to approximately optimize Eq. (8) among peptides of any fixed length. We find that the length for which Eq. (10) is largest among those with $f(e) > b$ is length $b-1$. This is because, for any fixed $\hat{\boldsymbol{\theta}}$, there is always an amino acid class for which $\hat{\eta}_i^j \geq 1$, and so adding one more amino acid from this class improves Eq. (8). To allow searching over hits of many different lengths, we instead use a probability distribution over lengths, which we take to be uniform, randomly sample a length, and construct of peptide with this length. This includes searching over peptides of length $b-1$ as a special case. We summarize the algorithm below in Algorithm 1.

This algoritm produces a set S of sequences e of amino acid classes. For each position within each of these amino acid class sequences e, we randomly sample an amino acid from that class according to a uniform distribution to finalize the set of peptides to test.

We also describe two modifications of Algorithm 1. In the first modification, we may replace the point estimation step (step 6 by sampling the $\hat{\boldsymbol{\theta}}^{0,j}$ and $\hat{\boldsymbol{\theta}}^{1,j}$ from their posterior distribution. We run this modified step along with steps 7 and 9 repeatedly within an inner loop to obtain a collection of amino class sequences e. For each position, we then choose the most frequently represented class at that position within this collection. This is the sequence of amino acid classes that we then add to S in step 10. This approach is less computationally intensive that implementing the greedy algorithm exactly, but uses more distributional estimation than Algorithm 1. We refer to this modification as "sampling-based" POOL, and to the version setting $\hat{\boldsymbol{\theta}}^{y,j}$ to the posterior mean as "mean-based" POOL.

In the second modification, instead of adding a single copy of e to S in step 10, we add multiple copies. This pushes POOL's future recommendations further away from its past recommendations, and encourages more diversity. We refer to this modification as "add-k", where k refers to the number of copies added, and "add-1" corresponds to unmodified POOL.

Algorithm 1 Greedy algorithm implementation

procedure GREEDY(k, J, K, dataset $\mathcal{D} = \{(\mathbf{x}^1, y^1), \ldots, (\mathbf{x}^N, y^N)\}$ and prior hyperparameters $\boldsymbol{\alpha}^{y,j}$)
  $S \leftarrow \emptyset$
  Calculate posterior distribution of $\boldsymbol{\theta}^{1,j} \sim \text{Dirichlet}(\boldsymbol{\theta}^{1,j} \mid \{\mathbf{x} : \mathbf{x} \in \mathcal{D}, y(\mathbf{x}) = 1\})$.
  for $m = 1$ to k **do**
    Calculate the posterior distribution of $\boldsymbol{\theta}^{0,j}$ and $\boldsymbol{\theta}^{1,j}$ given the dataset $\mathcal{D}$ and that $y(\mathbf{x}) = 0$ for all $\mathbf{x} \in S$.
    Calculate point estimators (either the posterior mode or the posterior mean) $\hat{\boldsymbol{\theta}}^{1,j}$ and $\hat{\boldsymbol{\theta}}^{0,j}$ of $\boldsymbol{\theta}^{0,j}$ and $\boldsymbol{\theta}^{1,j}$ for all j.
    Let $\hat{\eta}_i^j = \frac{\hat{\theta}_i^{1,j}}{\hat{\theta}_i^{0,j}}$ for all i and j.
    Choose a desired length at random from a distribution over lengths smaller than b.
    Construct $\mathbf{x}$ by setting $x_j = \arg\max_i \hat{\eta}_i^j$ for each j up to the desired length. Let e be the corresponding sequence of peptide classes.
    $S \leftarrow (S, e)$.

H. Using partial labeling information. When searching for specific hits, $y(e)$ is determined by considering several separately observable labels. For example, when searching for Sfp-specific hits, to know whether a peptide is a hit, we must observe both whether a peptide is a substrate for Sfp and for AcpS. If we have data for only one enzyme, then we do not observe $y(e)$. While we could simply discard such observations, this is not an efficient use of information. Here we describe how to use the POOL methodology when searching for specific hits while using measurements of less than the full set of enzymes for some training data.

Let $y_i(e)$ be a binary label for desired constituent property i, so that $y(e) = \prod_i y_i(e)$. For example, when searching for Sfp-specific hits, let $y_1(e) = 1$ when e is a substrate for Sfp and 0 otherwise, and let $y_2(e) = 1$ when e is not a substrate for AcpS and 0 otherwise. Then $y(e) = 1$ can only happen when e is a substrate for Sfp and not for AcpS.

To utilize as much information as possible, instead of building Naive Bayes classifier that predicts $y(e)$ directly, we build separate Naive Bayes classifiers that predict each $y_i(e)$. Then, we may follow the same reasoning with which we derived the POOL algorithm (maximizing probability of improvement using a greedy approach), and see that we can create a set of peptides to test by iteratively adding to S the peptide that maximizes Eq. (8).

Using the property that $y(e) = \prod_i y_i(e)$, we may rewrite Eq. (8) as

$$\arg\max_{e \in E \setminus S, f(e) > b} \mathbb{P}(y_i(e) = 1, \forall i \mid \prod_i y_i(e') = 0, \forall e' \in S). \qquad [11]$$

This is hard to maximize exactly because the number of configurations of $(y_i(e') : i, e' \in S)$ that satisfy $\prod_i y_i(e') = 0$ for all e' grows exponentially with the size of S, and computing Eq. (11) quickly becomes infeasible as S grows. We instead solve Eq. (11) using a heuristic approach in which we approximate the objective by

$$\mathbb{P}\left(y_i(e) = 1, \forall i \mid y_i(e') = 0, \forall i, \forall e' \in S\right). \qquad [12]$$

This can be optimized efficiently using an approach analogous to the one in Algorithm 1, but where we condition on $y_i(e) = 0, \forall i$ whenever that algorithm conditions on $y(e) = 0$.

Training sets 1-3 were generated using a single machine learning model. Training sets 4 and 5 were generated using partial labeling information.

3. Simulation-based comparison of POOL with existing methods

Figure 3 shows results of a simulation study in which we compared POOL with two existing methods: the predict-then-optimize or pure-exploitation method; and the mutation method. Here we define these two methods precisely (Sections A), provide a more detailed discussion of the predict-then-optimize method (Section B) and describe the method used embed peptide sequences in two-dimensional space in Figure 3 C-D (Section C).

A. Definitions of existing methods. In our simulation study we compare with the predict-then-optimize method and the mutation method:

- In the predict-then-optimize method, we rank peptides that have not been tested according to $P(y(e) = 1)$, as calculated by the Naive Bayes predictive model. Then, if we are allowed to recommend k peptides to test, we recommend the k with the largest probabilities.

- In the mutation method, we choose a known hit at random and then mutate it as follows. We randomly select the number of positions to mutate from a uniform distribution over $(1, 2, 3, 4)$. We then randomly select this number of positions to mutate on the target peptide, uniformly and without replacement. Finally for each position chosen to mutate, we randomly choose an amino acid to replace the original amino acid, uniformly from among all 20 amino acids. (Note that with probability 1/20, this amino acid will be the same as the original.)

B. Discussion of predict-then-optimize. The predict-then-optimize approach does not consider how future activity measurements will change the machine learning model's predictions, and how in turn this should affect the order in which peptides are tested. We argue that this makes the approach unnecessarily brittle to inaccuracies in predictions, and causes the set of peptides tested to lack diversity. Indeed, if the first peptide tested by the predict-then-optimize approach is not a hit, then the second peptide is typically quite similar, and is likely to also not be a hit.

By considering how measuring one peptide's activity will change the prediction of another's activity, POOL performs a more complete accounting of the value of testing a particular set of peptides, which causes it to recommend a more diverse

set of peptides. We argue that this leads POOL to provide more robust performance, allowing it to find high-fitness active peptides in fewer experiments than the predict-then-optimize approach.

In this section, we illustrate this with an example. Suppose there are three peptides, A, B and C; A and B are very similar to each other, and their predicted probabilities of being a hit are both 0.9, written as $\mathbb{P}(A \text{ is a hit}) = \mathbb{P}(B \text{ is a hit}) = 0.9$, while C is different from A or B, and C has a lower predicted probability being a hit, say $\mathbb{P}(C \text{ is a hit}) = 0.8$. Also suppose that the events that A is a hit and B is a hit are perfectly correlated (since A and B are very similar), and are both independent from the event that C is a hit (since C is different from A and B). We also assume $f(A), f(B), f(C)$ are all greater than b.

We wish to find a hit by testing only test two peptides. Predict-then-optimize will test A and B, because they have higher predicted probabilities of being a hit. However, we will see that this choice does not provide the highest probability-of-improvement.

We compute the probability-of-improvement when choosing A and B as:

$$\begin{aligned}\mathrm{PI}(\{A,B\}) &= \mathbb{P}(A \text{ is a hit}, B \text{ is a hit}) \\ &+ \mathbb{P}(A \text{ is a hit}, B \text{ is not a hit}) \\ &+ \mathbb{P}(A \text{ is not a hit}, B \text{ is a hit}), \\ &= 0.9 + 0 + 0, \\ &= 0.9.\end{aligned}$$

However, the probability-of-improvement when choosing A and C is

$$\begin{aligned}\mathrm{PI}(\{A,C\}) &= \mathbb{P}(A \text{ is a hit}, C \text{ is a hit}) \\ &+ \mathbb{P}(A \text{ is a hit}, C \text{ is not a hit}) \\ &+ \mathbb{P}(A \text{ is not a hit}, C \text{ is a hit}), \\ &= 0.9 \times 0.8 + 0.9 \times 0.2 + 0.1 \times 0.8, \\ &= 0.98.\end{aligned}$$

Similarly, $\mathrm{PI}(\{B,C\}) = 0.98$. Therefore maximizing probability-of-improvement will result in testing either $\{A,C\}$ or $\{B,C\}$.

To understand this intuitively, suppose that the first peptide tested, A, is not a hit. Then B is also unlikely to be a hit because A and B are very similar to each other. On the other hand, since C is different than A and B, C may be a hit even if A and B are not. Therefore, choosing diversified and good peptides to test, i.e., choosing $\{A,C\}$ or $\{B,C\}$ in this example, is a better strategy than choosing peptides with high predicted value regardless of diversity, like choosing $\{A,B\}$. This diversity is the key to finding hits when the machine learning model prediction is not accurate.

C. Two-dimensional embedding of peptide sequences. Figure 3 C-D visualizes peptide sequences as points in two-dimensional space. To create this visualization, we first compute a distance between each pair of peptides included in the visualization as follows: First, we set three penalty parameters $\alpha = 10$, $\beta = 1.5$ and $\gamma = 1$. Then, we align the two sequences at the central Serine. For each position to the left and right of the Serine, we then compute a penalty. If the two sequences have the same amino acid the penalty is 0. If the amino acids are different but are from the same class according to Supplementary Figure 2, then a penalty β is incurred. If the amino acids are from different classes then a penalty α is incurred. If one sequence has an amino acid at this position while the other does not, then a penalty γ is incurred. This penalty is then summed across all positions to the create the distance.

This method creates a matrix containing a distance between each pair of peptides. We then pass this matrix to the t-Distributed Stochastic Neighbor Embedding (t-SNE) method [11], implemented in Python in the scikit learn package (http://scikit-learn.org/stable/modules/generated/sklearn.manifold.TSNE.html). This produces a 2-dimensional point which we then plot for each peptide such that the distance between each pair of points approximates the distances provided in the matrix.

4. Normalization of peptide intensities between rounds and discussion of lead peptide determination

A. Analysis of peptide hits. After treatment by PPTases and AcpH, the fluorescent membranes were imaged by a Typhoon FLA 9500 variable mode laser scanner (GE Healthcare). The following settings were used to image the membranes: 50 µm resolution (50 px), 350V PMT, and Ex 532 nm/Em 580 nm.

B. Normalization calculation. Due to the change of light conditions and machine calibration, the readings of light intensity scale differently in different rounds and across membranes. We develop a normalization method that we apply to the raw data to allow comparing measurements across multiple rounds and membranes to identify peptide hits.

To support describing this normalization procedure, we define notation: We performed 5 rounds of experiments, and therefore j is one of 1,2,3,4, or 5. We also have 4 treatments: Sfp (k=1), Sfp + AcpH (k=2), AcpS (k=3), and AcpS + AcpH (k=4). For peptide i tested in round j for treatment k, we indicate the raw reading by $y_{i,j,k}$. We will indicate the corresponding "normalized" value by $\theta_{i,k}$.

We assume the raw reading y_{ijk} is the result of scaling and shifting the normalized value $\theta_{i,k}$ and adding noise:

$$y_{i,j,k} = \sigma_{j,k}\left(\theta_{i,k} + \epsilon_{i,j,k}\right) + \mu_{j,k},$$

where $\sigma_{j,k}$ is the scaling factor, $\mu_{j,k}$ is the shifting factor, and $\epsilon_{i,j,k}$ is independent normally distributed noise with mean 0 and variance a^2. In addition, we know that after AcpH treatment, the normalized light intensity is the same or lower than before treatment, therefore $\theta_{i,1} \geq \theta_{i,2}$ and $\theta_{i,3} \geq \theta_{i,4}$.

We fit the parameters $\mu_{j,k}, \sigma_{j,k}, \theta_{i,k}$ by maximizing the log-likelihood of the data, where the log-likelihood is

$$\log(L) = -\frac{1}{2}\sum_{i,j,k}\frac{1}{a^2}\left(\frac{y_{i,j,k} - \mu_{j,k}}{\sigma_{j,k}} - \theta_{i,k}\right)^2 + \frac{N}{2}\log(2\pi a),$$

and we enforce constraints when maximizing of $\theta_{i,1} - \theta_{i,2} \geq 0$ and $\theta_{i,3} - \theta_{i,4} \geq 0$ for all i. In addition, membranes include control peptides that are known to not react with AcpH after reacting with Sfp or AcpS. We set $\theta_{i1} - \theta_{i2} = 0$ for all $i \in I_1$, where I_1 is the set of peptides that are known to react with Sfp and not with AcpH, and $\theta_{i,3} - \theta_{i,4} = 0$, for all i in I_2, where I_2 is the set of peptides that are known to react with AcpS and not with AcpH. We additionally add the constraint that $\sum_i \theta_i$

should be equal to the constant 1, which avoids degenerate solutions in which $\sigma_{j,k}$ grows to ∞ while $\theta_{i,j}$ shrinks to 0.

Using the notation $\vec{\mu}$ to indicate the vector comprised of $\mu_{j,k}$ for all j and k, and similarly for $\vec{\sigma}$ and $\vec{\theta}$, we can write this fitting procedure more compactly as solving the following optimization problem.

$$\min_{\vec{\mu},\vec{\sigma},\vec{\theta}} \sum_{i,j,k} \left(\frac{y_{i,j,k} - \mu_{j,k}}{\sigma_{j,k}} - \theta_{i,k} \right)^2$$

$$\text{subject to:} \theta_{i,1} - \theta_{i,2} \geq 0$$

$$\theta_{i,3} - \theta_{i,4} \geq 0$$

$$\theta_{i,1} - \theta_{i,2} = 0 \text{ for all } i \in I_1$$

$$\theta_{i,3} - \theta_{i,4} = 0 \text{ for all } i \in I_2$$

$$\sum_i \theta_i = 1.$$

The problem above is a quadratic program, and can be solved efficiently by quadratic programming software such as Gurobi [12]. The solution gives us the normalized value $\theta_{i,k}$ for every peptide i and every treatment k.

5. Synthesis and Chemoenzymatic Synthesis

A. Synthesis of TAMRA-C6-Pantethenamide.

General Methods: Unless otherwise noted, all reagents and chemical compounds were purchased from Alfa Aesar, Sigma-Aldrich, Fisher Scientific or AAPPTec and used without further purification. Flash chromatography was carried out on 239-400 mesh grade 60 silica gel (Fisher Scientific). NMR spectra were recorded on Varian VX500 spectrometer. FID files were processed through using MestRenova (MestreLab Research). Mass spectrometric analyses were conducted on the ACQUITY ultra performance LC (Waters) with 2.6 μm, C18 100 Å, Kinetex LC column (150 x 3 mm).

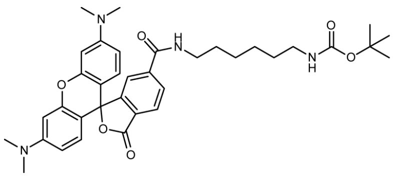

TAMRA-C6-Boc (1): Tert-butyl-(6-(3',6'-bis(dimethylamino)-3-oxo-3H-spiro[isobenzofuran-1,9'-xanthene]-6-carboxamido)hexyl)carbamate (**1**). 5-TAMRA was made according to literature procedures [13]. To a stirred solution of 5-TAMRA (300 mg, 0.69 mmol) in dry DMF (3 mL), HATU (300mg, 0.84 mmol) and DIPEA (250 µL, 1.35mmol) were added. The solution was stirred under nitrogen at room temperature for 30 minutes. *N*-Boc-1,6-diaminohexane (180mg, 0.84 mmol) was then added and the solution was left to stir overnight under nitrogen gas. The solution was then concentrated to dryness *in vacuo* then resuspended in 100mL of DCM and washed with aq $NaHCO_3$ (sat.) 3 x 50mL and brine 50 mL. The organic layer was dried over anhydrous Na_2SO_4 and then concentrated to dryness *in vacuo*. The residue was then run on silica gel in acetone to afford the product 247 mg (56.9%) as a purple solid. ^{1}H NMR (500 MHz, $CDCl_3$) : δ= 8.40 (bs, 1H), 8.20 (d, 1H, J= 8.1 Hz), 7.25 (d, 1H, J= 8.1 Hz), 6.59 (d, 2H, J= 8.8 Hz), 6.49 (d, 2H, J= 2.6 Hz), 3.48 (q, 2H, J= 6.0 Hz), 3.13 (m, 2H), 3.00 (s, 12H), 1.66 (m, 2H), 1.49 (m, 4H) 1.44 (s, 9H), 1.36 (m, 2H) ^{13}C NMR (125 MHz, $CDCl_3$): δ=169.13, 165.94, 156.09, 153.01, 152.25, 136.32, 134.12, 128.70, 124.69, 122.86, 108.79, 106.26, 98.37, 79.03, 40.19, 40.00, 39.75, 29.97, 29.25, 28.37, 26.06, 25.82. HR-MS $[M+H]^+$ Theo: 629.3334 Obs: 629.3330.

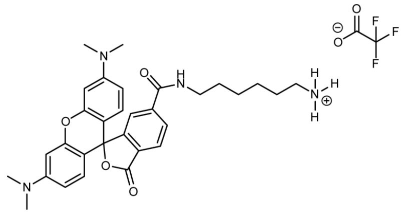

TAMRA-C6-NH$_3^+$ (2): 6-(3',6'-bis(dimethylamino)-3-oxo-3H-spiro[isobenzofuran-1,9'-xanthene]-6-carboxamido)hexan-1-ammonium TFA Salt (**2**). TAMRA-C6-BOC (**1**) (150 mg) was stirred in 10 mL of TFA for 2 hours. The solution was poured into 100 mL of diethyl ether and the precipitate was filtered and dried *in vacuo* to give 120 mg of the product as a purple solid (99%). ^{1}H NMR (500 MHz, DMSO-d$_6$) : δ= δ 9.00 (t, J = 5.6 Hz, 0H), 8.72 (d, J = 1.8 Hz, 0H), 8.34 (dd, J = 7.8, 1.8 Hz, 0H), 7.94 (s, 1H), 7.61 (d, J = 7.9 Hz, 0H), 7.08 (dt, J = 15.0, 5.7 Hz, 2H), 6.95 (d, J = 2.2 Hz, 1H), 3.37 (q, J = 6.6 Hz, 1H), 3.28 (s, 6H), 2.84 (h, J = 5.9 Hz, 1H), 1.61 (h, J = 6.8 Hz, 2H), 1.40 (p, J = 3.7 Hz, 2H). ^{13}C NMR (125 MHz, DMSO-d$_6$) δ 166.45, 165.04, 159.10, 158.85, 158.59, 158.34, 157.18, 157.08, 136.60, 131.77, 131.59, 131.01, 130.80, 129.95, 118.58, 116.24, 115.00, 113.10, 96.71, 40.98, 40.61, 39.24, 29.38, 27.50, 26.55, 26.08. HR-MS $[M+H]^+$ Theo: 529.2804 Obs: 529.2810

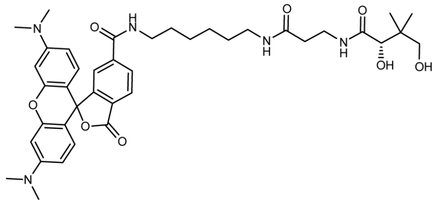

TAMRA-C6-Pantethenamide (3): (S)-N-(6-(3-(2,4-dihydroxy-3,3-dimethylbutanamido)propanamido)hexyl)-3',6'-bis(dimethylamino)-3-oxo-3H-spiro[isobenzofuran-1,9'-xanthene]-6-carboxamide (**3**). TAMRA-C6-NH$_3^+$ (**2**) (100 mg) stirred in 2 mL of DMF with 200 mg of HATU and 150 mg of PMB protected pantothenic acid. The solution was poured into 50 mL of DCM and washed with 3 x 25 mL of saturated sodium bicarbonate and 25 mL of brine. The organic layer was concentrated *in vacuo*. The residue was then dissolved in 5 mL of 80% acetic acid in water. The solvent was removed via rotary evaporator and the crude mixture was run on preparative HPLC 25% ACN/H_2O to 40% ACN/H_2O over 50 minutes retention time (26 minutes). Fractions containing the product were combined and lyophilized to give 60 mg (69%) of the product as a light purple solid. ^{1}H NMR (500 MHz, DMSO-d$_6$) : δ= 8.92 (t, J = 5.7 Hz, 0H), 8.69 (d, J = 1.8 Hz, 0H), 8.30 (dd, J = 8.0, 1.8 Hz, 0H), 7.98 – 7.88 (m, 1H), 7.57 (d, J = 7.9 Hz, 0H), 7.10 – 6.99 (m, 2H), 6.93 (d, J = 2.1 Hz, 1H), 3.70 (s, 0H), 3.38 – 3.26 (m, 2H), 3.25 (s, 6H), 3.26 – 3.13 (m, 1H), 3.03 (qd, J = 6.9, 2.7 Hz, 1H), 2.26 (td,

J = 7.0, 2.2 Hz, 1H), 1.56 (q, J = 7.1 Hz, 1H), 1.40 (p, J = 7.1 Hz, 1H), 1.38 – 1.26 (m, 2H), 0.78 (d, J = 14.5 Hz, 3H). ^{13}C NMR (125 MHz, DMSO-d$_6$): δ=173.28, 170.72, 166.40, 164.95, 158.59, 158.33, 157.21, 157.10, 136.58, 136.26, 131.73, 131.54, 131.03, 130.84, 129.99, 115.04, 113.15, 96.67, 75.35, 75.34, 68.45, 40.96, 39.49, 38.85, 35.56, 35.28, 29.57, 29.47, 26.71, 26.67, 21.38, 20.71. HR-MS $[M+H]^+$ Theo: 729.3738 Obs: 730.3808.

B. Chemoenzymatic Synthesis of TAMRA Coenzyme A (CoA).

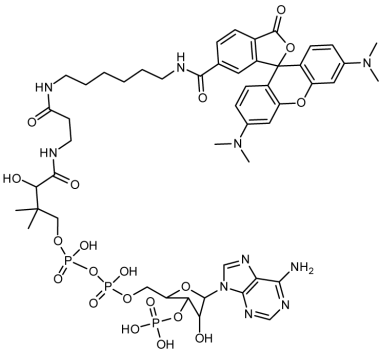

TAMRA-CoA (4). Compound **4** was synthesized *in situ* using CoA biosynthetic enzymes (CoaA, CoaD, CoaE) as previously reported [14, 15]. The 1 mL reaction volume was incubated at 37°C shaking for 8-12 hours and contained 5 mM of TAMRA-C6-Pantethenamide (**3**), 32 mM ATP pH 8, 0.01 μg μL^{-1} *S. aureus* 6-His CoaA, 0.01 μg μL^{-1} *E. coli* MBP CoaD, and 0.01 μg μL^{-1} *E. coli* MBP-CoaE in 50 mM Tris/HCl buffer pH 7.5. The lyophilized reaction mixture was purified on a VyDAC protein & peptide C18 300 Å (4.6 x 250 mm) column using an Agilent 1100 semi-preparative HPLC using a reverse-phase protein peptide C18 column with a solvent system of (A) 0.1% TFA in water and (B) 0.1% TFA in acetonitrile at 1 ml min^{-1}. The run consisted of 95% of buffer A for 5 minutes, followed by a gradient of 95% buffer A to 50% buffer A over 30 minutes. TAMRA-CoA was monitored at 254 nm. Purified compound **4** was lyophilized and identity confirmed by HRMS: $C_{50}H_{65}N_{10}O_{20}P_3$ 1219.03.

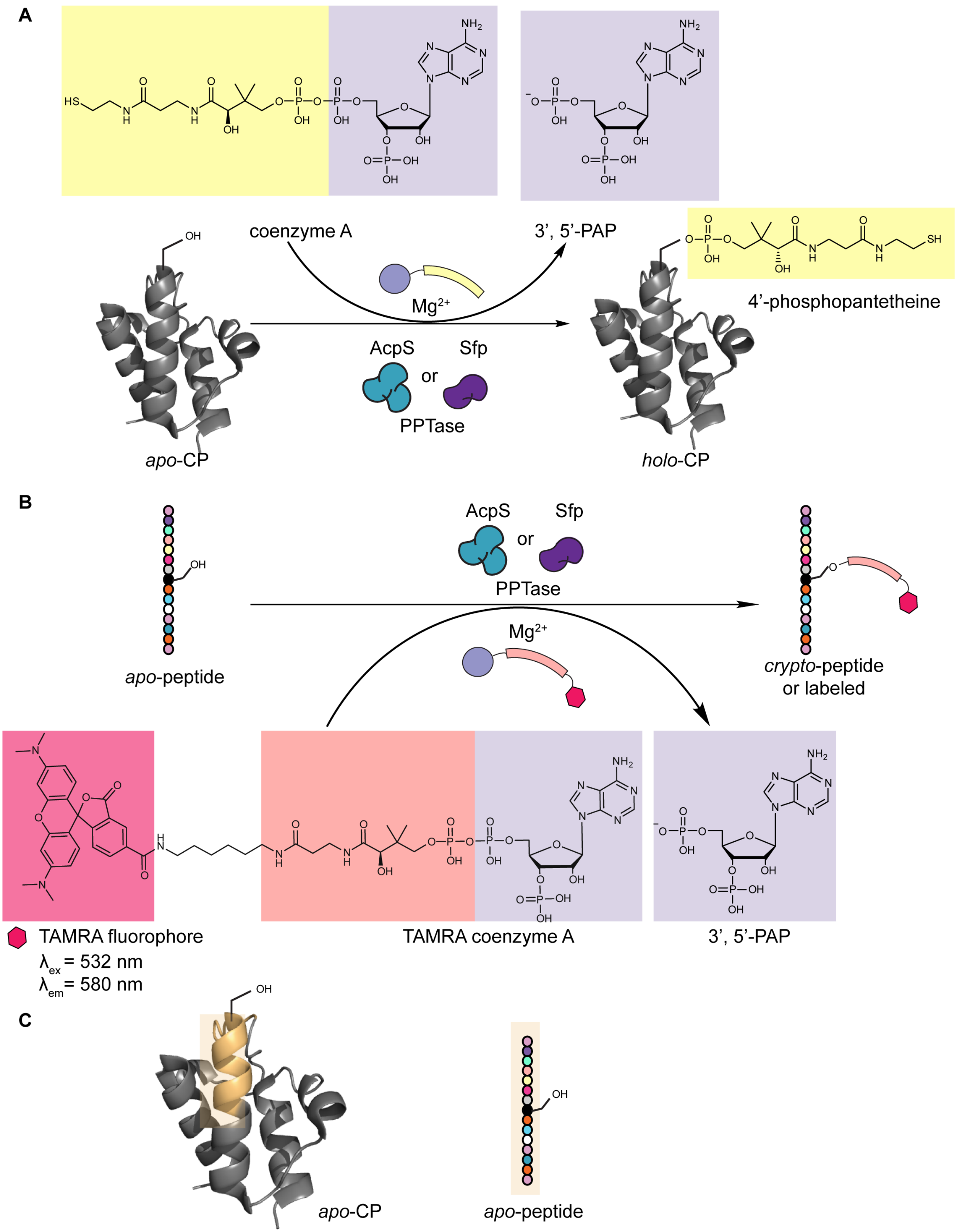

Supplementary Figure 1. (A) Reaction scheme illustrating labeling by PPTase of carrier protein with conserved serine. PPTase: 4'-phosphopantetheine transferase; CP: carrier protein. (B) Reaction scheme illustrating labeling by PPTase of POOL peptide substrate at conserved serine with TAMRA-CoA analog. (C)The *apo*-peptide is designed to act as a surrogate for the second helix (highlighted in orange) of the carrier protein containing the serine modification site.

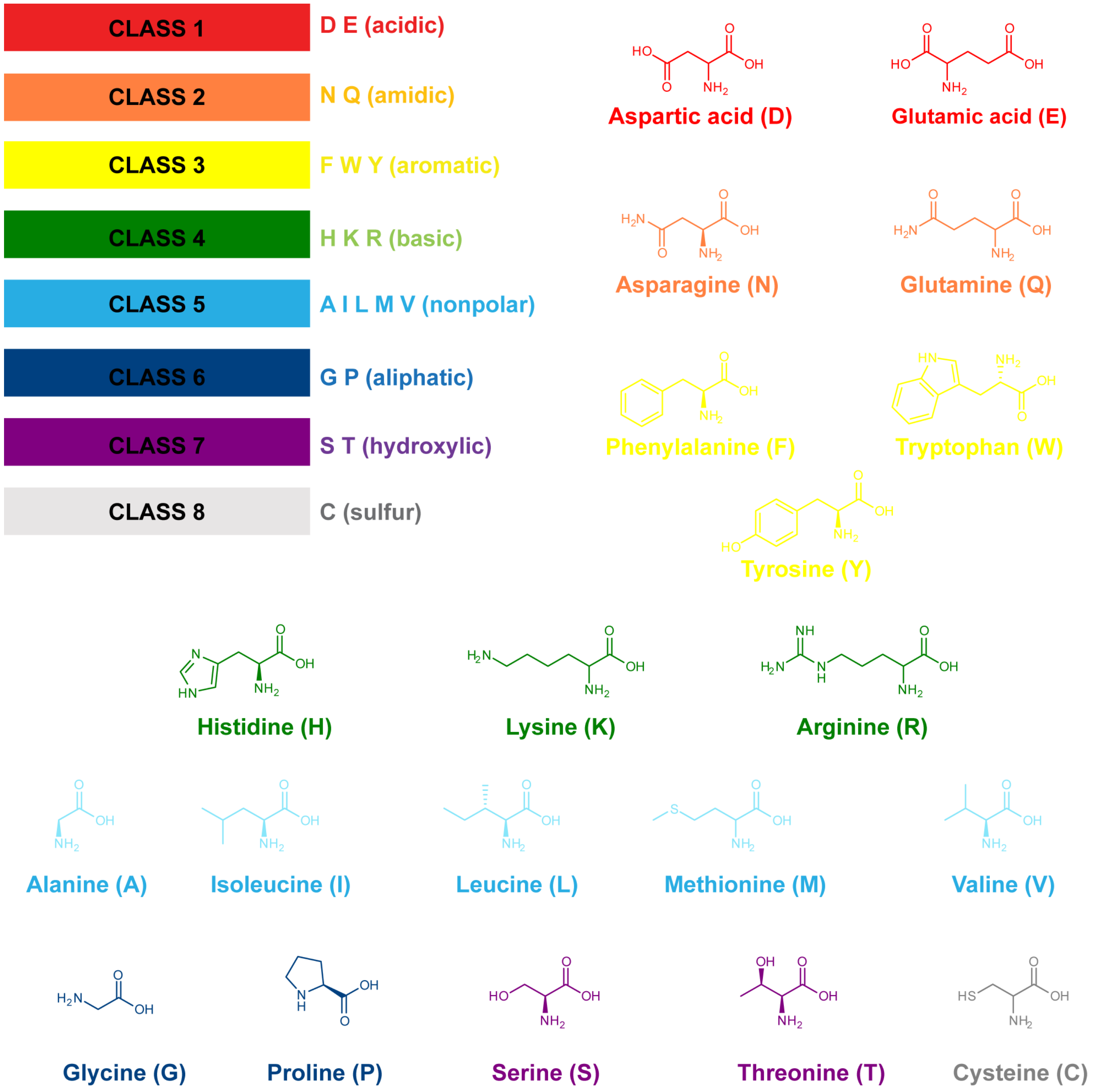

Supplementary Figure 2. Twenty essential amino acids organized by 8 reduced classes.

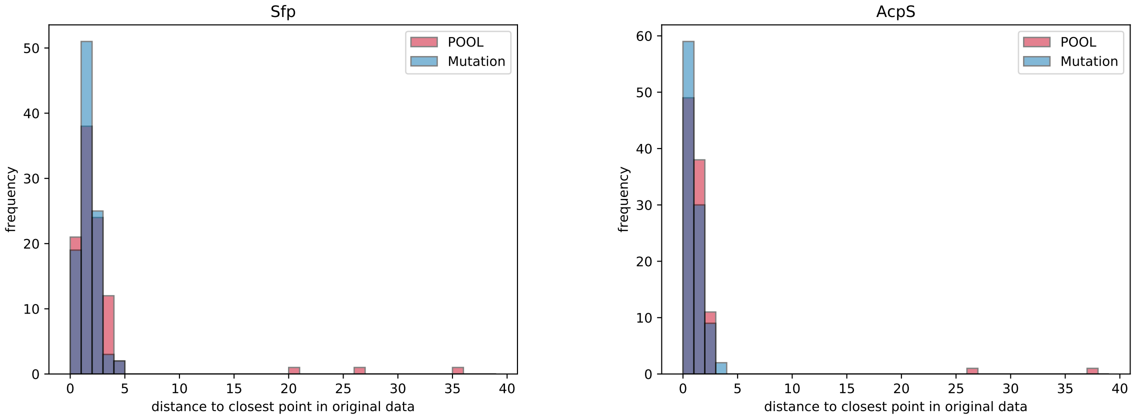

Supplementary Figure 3. Distances in simulation study between training data and peptides recommended by POOL and Mutation: As discussed in the main text ("Diversity of peptides recommended in the simulation study") and our discussion of this simulation study (Section 3), peptides recommended by the Mutation method tend to be closer to the training data than those recommended by POOL. Recall that our simulation study represents peptides by 2-dimensional points. We calculated the Euclidean distance in 2-dimensions between the closest point in the training data and each peptide recommended by POOL and Mutation. The left plot shows the distributions of these distances for the two methods when searching for Sfp-specific peptides; the right plot shows the distributions when searching for AcpS-specific peptides. In each plot, two overlapping histograms are shown, where red indicates POOL, blue indicates Mutation, and purple indicates an overlap in the two histograms. We also provide summary statistics from these histograms. For the left (Sfp) plot: mean 1.63 (Mutation), 2.55 (POOL); median 1.58 (Mutation), 1.64 (POOL); 25% quartile 1.12 (Mutation), 1.13 (POOL); 75% quartile 2.08 (Mutation), 2.57 (POOL). For the right (AcpS) plot: mean 0.97 (Mutation), 1.67 (POOL); median 0.86 (Mutation), 1.01 (POOL); 25% quartile 0.40 (Mutation), 0.64 (POOL); 75% quartile 1.22 (Mutation), 1.37 (POOL).

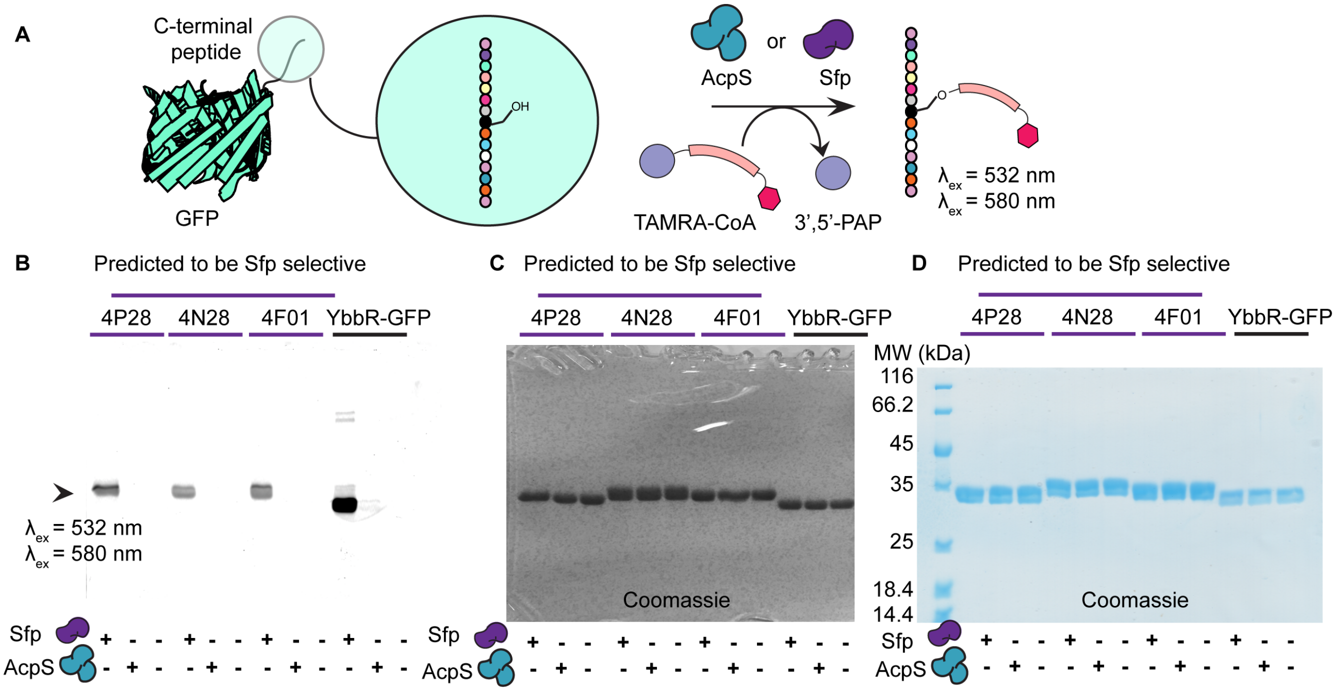

Supplementary Figure 4. Full SDS-PAGE images of GFP-peptide fusion proteins after PPTase labeling. (A) Repeat image for clarity of illustrating Sfp labeling of GFP-peptide fusion proteins from main text Fig. 5A. (B-C) Full 12% SDS-PAGE gel images corresponding to the main text Fig. 5B. (D) An additional Coomassie gel containing a molecular weight marker was run to confirm the correct size of each GFP-peptide fusion. PPTase (Sfp and AcpS) is not observed due to low abundance (Coomassie sensitivity). TAMRA-CoA was added to all reactions including no enzyme control.

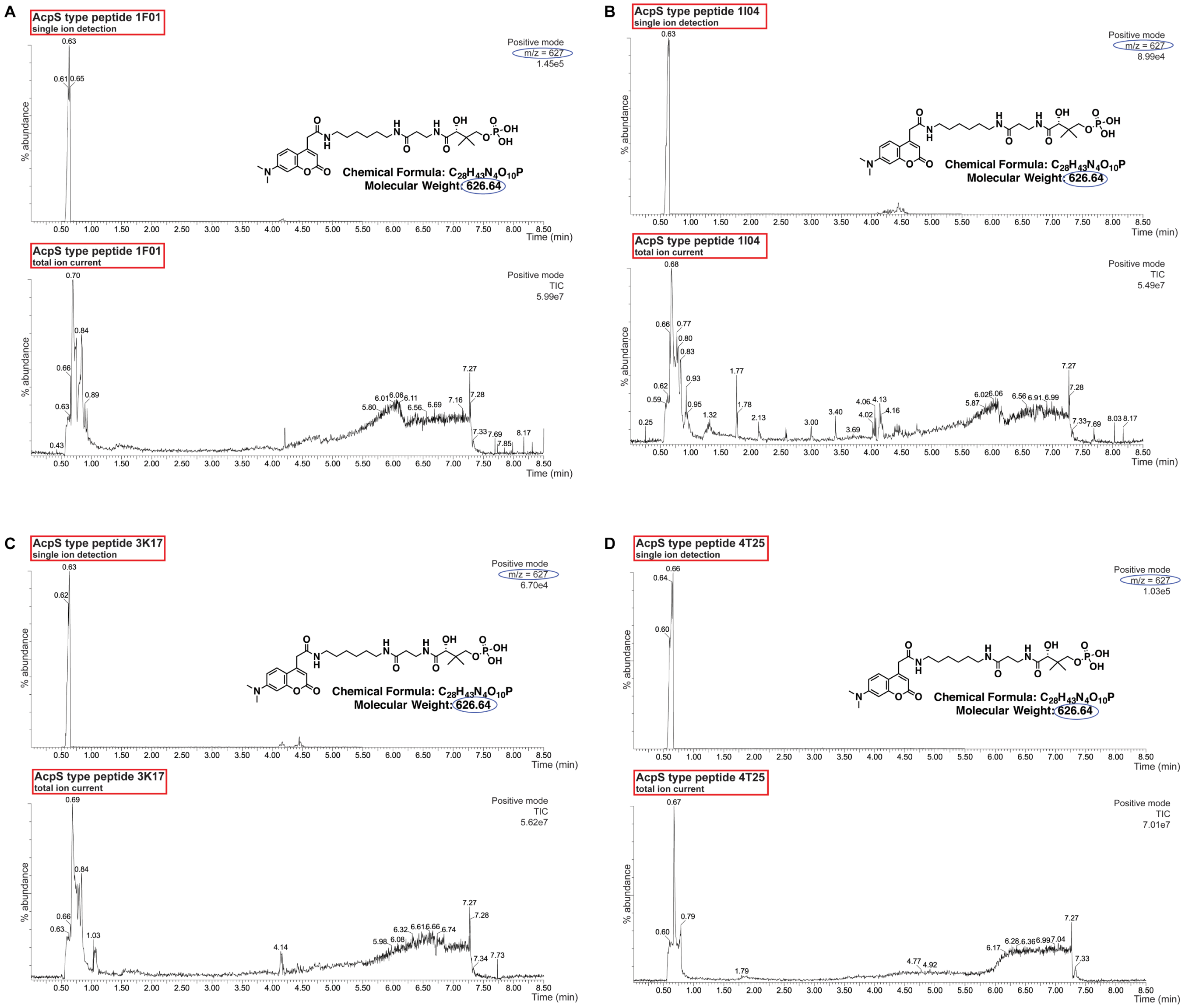

Supplementary Figure 5. LC-MS for characterizing labeling of AcpS peptides: *In cellulo* labeling of coumarin CoA onto GFP-peptide by endogenous *E. coli* PPTase (AcpS) was confirmed by LC-MS analysis of coumarin phosphopantetheine after hydrolysis from the GFP-peptide [16]. (A) LC-MS analysis of hydrolyzed phosphopantetheine from AcpS-type peptide 1F01 was monitored by single ion detection mode set for measuring M+1=627, coumarin phosphopantetheine : m/z=626 (shown above). LC-MS trace showing total ion current of the same sample (shown below). (B) LC-MS analysis of hydrolyzed phosphopantetheine from AcpS-type peptide 1I04 was monitored by single ion detection mode (shown above). LC-MS trace showing total ion current of the same sample (shown below). (C) LC-MS analysis of hydrolyzed phosphopantetheine from AcpS-type peptide 3K17 was monitored by single ion detection mode (shown above). LC-MS trace showing total ion current of the same sample (shown below). (D) LC-MS analysis of hydrolyzed phosphopantetheine from AcpS-type peptide 4T25 was monitored by single ion detection mode (shown above). LC-MS trace showing total ion current of the same sample (shown below).

GFP FUSION PEPTIDE ID	Peptide Sequence	k_{cat} min^{-1}	K_m μM	k_{cat} / K_m $\mu M \cdot min^{-1}$
YbbR-GFP	D S L E F I A S K L A	2.8 ± 0.2	0.5 ± 0.1	5.9 ± 1.5
**4P28**	L I G I D S I E T L K A	0.20 ± 0.02	0.4 ± 0.2	0.5 ± 0.1
**4N28**	Y G D E I P A E S L D F L E	0.04 ± 0.01	1.6 ± 0.6	0.03 ± 0.01
**4F01**	A V K M E S L E Y L D T M	1.3 ± 0.1	0.6 ± 0.1	2.2 ± 0.6

Supplementary Table 1. Gel-based electrophoretic mobility shift kinetics for *B. subtilis* Sfp

Supplementary References

1. Yin, J. *et al.* Genetically encoded short peptide tag for versatile protein labeling by Sfp phosphopantetheinyl transferase. *Proc. Natl. Acad. Sci. U. S. A.* **102**, 15815–15820 (2005).

2. Dall'Aglio, P. *et al.* Analysis of *streptomyces coelicolor* phosphopantetheinyl transferase, AcpS, reveals the basis for relaxed substrate specificity. *Biochemistry* **50**, 5704–5717 (2011).

3. Kosa, N. M., Pham, K. M. & Burkart, M. D. Chemoenzymatic exchange of phosphopantetheine on protein and peptide. *Chem. Sci.* **5**, 1179–1186 (2014).

4. Lewis, D. D. Naïve (Bayes) at forty: The independence assumption in information retrieval. In *Machine Learning: ECML-98*, 4–15 (Springer, Berlin, Heidelberg, 1998).

5. Howard, R. A. Information value theory. *IEEE Transactions on Systems Science and Cybernetics* **2**, 22–26 (1966).

6. Frazier, P. I. Decision-theoretic foundations of simulation optimization. *Wiley Encyclopedia of Operations Research and Management Science* (2010).

7. Lewis, D. D. & Gale, W. A. A sequential algorithm for training text classifiers. In Croft, B. W. & van Rijsbergen, C. (eds.) *SIGIR '94*, 3–12 (Springer, London, 1994).

8. McCallum, A. & Nigam, K. A comparison of event models for naïve Bayes text classification. In *AAAI-98 Workshop on Learning for Text Categorization*, vol. 752, 41–48 (1998).

9. Nemhauser, G. L., Wolsey, L. A. & Fisher, M. L. An analysis of approximations for maximizing submodular set functions. *Math. Program.* **14**, 265–294 (1978).

10. Sorenson, H. W. H. W. *Parameter Estimation: Principles and Problems* (Marcel Dekker, 1980).

11. Maaten, L. v. d. & Hinton, G. Visualizing data using t-SNE. *J. Mach. Learn. Res.* **9**, 2579–2605 (2008).

12. Gurobi Optimization, Inc. Gurobi Optimizer. (http://www.gurobi.com/products/gurobi-optimizer) (2017).

13. Kvach, M. V. *et al.* Practical synthesis of isomerically pure 5-and 6-carboxytetramethylrhodamines, useful dyes for dna probes. *Bioconjugate Chem.* **20**, 1673–1682 (2009).

14. Nazi, I., Koteva, K. P. & Wright, G. D. One-pot chemoenzymatic preparation of coenzyme A analogues. *Anal. Biochem.* **324**, 100–105 (2004).

15. La Clair, J. J., Foley, T. L., Schegg, T. R., Regan, C. M. & Burkart, M. D. Manipulation of carrier proteins in antibiotic biosynthesis. *Chem. Biol.* **11**, 195–201 (2004).

16. Dorrestein, P. C. *et al.* Facile detection of acyl and peptidyl intermediates on thiotemplate carrier domains via phosphopantetheinyl elimination reactions during tandem mass spectrometry. *Biochemistry* **45**, 12756–12766 (2006).
